# The father’s singing voice may impact premature infants’ brain more than their mother’s: A NICU single-arm exploratory study protocol and preliminary data on a singing and EEG framework based on the fundamental frequency of voice and kinship

**DOI:** 10.1371/journal.pone.0328211

**Published:** 2025-08-14

**Authors:** Efthymios Papatzikis, Kyriakos Dimitropoulos, Kassandra Tataropoulou, Maria Kyrtsoudi, Elena Pasoudi, John M. O’Toole, Angeliki Nika

**Affiliations:** 1 Department of Early Childhood Education and Care, Oslo Metropolitan University, Oslo, Norway; 2 Advanced Health Intelligence and Brain-Inspired Technologies (ADEPT) Research Group, Oslo Metropolitan University, Oslo, Norway; 3 Neonatal Intensive Care Unit, ‘Panagiotis and Aglaia Kyriakou’ Children’s Hospital, Athens, Greece; 4 Department of Music Science and Art, University of Macedonia, Thessaloniki, Greece; 5 Clinical Psychoacoustics Laboratory, Medical School, Aristotle University of Thessaloniki, Thessaloniki, Greece; 6 Independent Researcher, Athens, Greece; 7 CergenX. Ltd, Dublin, Ireland; Air University, PAKISTAN

## Abstract

This article reports the protocol of a single-arm exploratory study investigating the impact of singing on the brain activity of premature infants in the Neonatal Intensive Care Unit (NICU). The study focuses on how the differentiation of voices, as defined by the fundamental frequency (F0) shaped by biological sex and kinship, influences neurophysiological responses when measured by electroencephalography (EEG). Premature infants, who are highly sensitive to auditory stimuli, may benefit from music-based interventions; however, there is limited understanding of how voice variations between male and female caregivers, and whether they are biologically related, affect brain activity. Our protocol outlines a structured intervention where infants are exposed to singing by four facilitators – a male and a female music therapist, the mother, and the father – and includes two singing stages: a sustained note and a lullaby, both interspersed with silent periods to allow for baseline measurements. EEG recordings track brain activity throughout these sessions, followed by quantitative EEG (qEEG) analysis and thorough statistical computations (e.g., mixed-effects models, spectral power analysis, and post-hoc tests) to explore how these auditory stimuli influence brain function. Preliminary data from five infants show that maternal singing elicits the highest delta spectral power in all measured conditions except during the ‘lullaby song’, where paternal singing elicits the highest effects followed by the male music therapist and then the mother. These early findings highlight the potential influence of parental voices, particularly the fathers’ voice, on neonatal brain development, while the detailed study protocol ensures rigor and replicability, providing a robust framework for future research. (clinicaltrials.gov unique identifier: NCT06398912).

## Introduction

In the evolving landscape of neonatal neurology, the focus has increasingly shifted towards understanding the complex interplay between the environment and the developmental outcomes of neonates, especially those in critical care settings [1–3]. Globally, a significant number of newborns (just over 13 million) are born prematurely, and many of these vulnerable infants spend their early, formative days in Neonatal Intensive Care Units (NICUs) [4]. Advances in neonatal medicine have dramatically improved the survival rates of these infants [e.g., 5, 6] yet challenges persist in ensuring their long-term neurological health and development. Emerging evidence indicates that prematurity is associated with a heightened risk for enduring neurological and cognitive impairments – including motor deficits, attention difficulties, and behavioral challenges – that may impact academic achievement and quality of life well into adolescence and adulthood [7,8].

Structured sound interventions, approached as non-pharmacological supplementary treatments, have shown promise in improving health outcomes for this vulnerable population in the context of the NICU. For example, white noise has been found to mimic the intrauterine environment, aiding in the stabilization of sleep patterns and reducing stress responses in premature infants [9]. The use of heartbeat sounds has also been instrumental in providing a rhythmic and familiar auditory stimulus to these infants, facilitating physiological regulation and promoting a sense of security [10,11]. Similarly, environmental sounds, such as gentle rain or soft wind, have been shown to create a soothing and more natural auditory environment, contributing to the reduction of the clinical and mechanical noise of the NICU [12–14]. Finally, music – the highest and most complex form of structured sounds, offered via music medicine or music therapy sessions (for a distinction between the two see [15]) – has been widely documented to enhance infants’ physiological and emotional well-being [16–18], with studies demonstrating the respective positive effects on stress reduction, pain management, and weight gain (for example see [19–22]).

### Delivering (live) music interventions in the NICU

While NICUs are pivotal for medical care, they also introduce infants, especially the premature ones, to unique sensory environments [23–25]. These environments differ significantly from the acoustical environment of the mother’s womb or the typical domestic environment, and can profoundly influence developmental processes [26–28]. Therefore, the prescription and implementation of music in NICUs should adhere to tailored and sensitive intervention protocols based on well-studied [29] and biology-based facts [30] to avoid disrupting the developmental trajectory of the infant and potentially act as a developmental catalyst.

For instance, regarding the administration of any sound stimulus in the NICU context, careful moderation is expected to prevent overstimulation and ensure the maintenance of physiological stability in neonates. The extreme sensitivity of premature infants to auditory stimuli necessitates that all sound related stimuli in the NICU should adhere to certain acoustical regulations as defined by specific regulatory medical bodies or global standards [31,32]. These regulations are designed not only to prevent adverse fluctuations in vital signs such as heart rate and respiration – ultimately resulting in stress responses in the infant – but also to optimize the therapeutic benefits of environmental and intentionally administered sound stimuli. Additionally, they aim to minimize potential disruptions to proper biomechanical development, such as damage to the hair cells in the infant’s inner ear. These specialized ear cells are very sensitive to apoptosis events and can take place due to excessive sound stimulation [33–35].

Furthermore, the timing of sound-related interventions should be strategically planned not only to align with each infant’s individual feeding and sleep-wake cycles [36] – as these factors can potentially interfere with the beneficial effects of musical interventions – but also to ensure they occur during quieter periods in the NICU, allowing for safe and effective delivery [14]. In particular, live singing interventions must be carefully calibrated and monitored to stay within safe sound limits set by medical regulations (see [32]). These regulations generally stipulate that sound levels outside the incubator should not exceed a continuous average of 45–50 dB, with transient peaks not surpassing 65 dB. Unfortunately, even highly trained professional singers, when attempting to maintain the lowest possible sound pressure levels (SPLs), may still exceed these limits. A study by Akerlund & Gramming [37] found that trained female singers could only produce minimum SPLs around 51.5 dB at low pitches, which already slightly exceeds the upper recommended continuous sound level in NICUs.

Finally, a seamless integration of any musical intervention into the infants’ overall care regimen requires close coordination with the medical team, as this collaborative effort is vital for aligning the intervention sessions with the already established medical protocols and tailoring them to meet the specific health requirements and medical conditions of each infant [38]. Such integration helps to ensure that the music intervention sessions are not only safe but also effectively support the broader therapeutic goals set forth by the healthcare providers, which especially in this sensitive and demanding in terms of physical development context require the parental involvement as another critical aspect according to the latest clinical research [39,40].

### The role of kinship in the NICU singing process

Prematurity poses significant risks to child development on social, behavioral, and cognitive levels [41]. Most importantly, the early bond between the biologically related caregiver and the infant is crucial for the latter’s cognitive development, influencing the child’s self-representation and future interpersonal relationships [42–43]. However, in the NICU, the typical biological processes of feeding, caring, and bonding are often disrupted due to the infant’s medical complexities, leading to stress for both infants and parents [44].

Parental-infant bonding issues are prevalent among parents who have undergone significant trauma, impairing their sensitivity towards their baby and heightening their vigilance about the baby’s condition [45–48]. Research indicates that premature infants often exhibit more passive behavior, less positive affect, and reduced facial expressions during their interactions with their biological caregivers [49]. Correspondingly, parents of premature infants tend to be less sensitive in these interactions, negatively impacting the infant’s developmental trajectory [50–51]. Conversely, sensitive caregiver attitudes and parent-infant synchronization during interactions have been shown to be protective and beneficial for the development of premature infants [51–53].

In this context, singing can serve as a parent-infant synchronization intervention, fostering emotional bonds and providing comfort to both infants and their parents [54–55]. Additionally, singing in the NICU can stabilize an infant’s physiological state, including heart rate and breathing, and promote better sleep patterns [56–57]. It can also serve as a conduit for emotional expression and connection, offering a sense of normalcy and control for parents who might otherwise feel helpless in the highly medicalized environment of the NICU. As a result, through singing, parents can engage in meaningful interaction with their infants, reinforcing their role as primary caregivers and fostering secure bonding and attachment [58–59].

However, parents are not the only caregivers in the NICU, and for this reason they are not the only ones who may be able to provide singing sessions. Multiple research studies have shown that caregivers with no immediate kinship – like nurses, physicians and other clinical staff – can also effectively use singing to support developmental and medical outcomes [60]. This dynamic is, of course, complex and influenced by factors such as the differentiation of kinship and the recognizability of the caregiver’s voice. The fundamental frequency of their voice, which differs from that of the mother and father, may be less familiar to the infant [61–63].

### The fundamental frequency of singing

In the context of the NICU, understanding the technical features of the singing voice, including its fundamental frequency (F0) as distinct from pitch, becomes increasingly vital, not only due to the auditory sensitivity of premature infants but also because of the developing nature of their central nervous system, which is highly susceptible to the variability of external stimuli as they grow older.

The fundamental frequency (measured in Hertz) refers to a physical property of sound waves – specifically, the number of vibrations per second produced, for example, by the violin string or, in our case, the vocal cords [64–65]. Pitch, on the other hand, is the perceptual correlate of fundamental frequency; it describes how the brain interprets and perceives the fundamental frequency [64]. While F0 is an objective measurement, pitch is a more subjective experience; however, for most periodic sounds, the fundamental frequency closely corresponds to the perceived pitch [64–65]. As a result, F0 is often used as a proxy for pitch in acoustic analysis and research.

Given the critical role of fundamental frequency (F0) in shaping the auditory environment for infants, particularly in a singing context, it is important to understand the differences in F0 between male and female voices. These differences, driven by anatomical factors such as the length of the vocal tract and the size of the laryngeal cavities, result in male voices typically having a lower F0 – around 100–120 Hz – compared to the higher F0 of female voices, generally in the 200–220 Hz range [66–68]. Such distinctions may influence how infants respond to structured sound stimuli, especially in light of the well-documented human sensitivity to voice discrimination based on biological sex [69]. The fundamental frequency serves as the foundation upon which harmonic structures are built, guiding the singing experience and creating a scaffold for the infant’s brain to process the embedded complex auditory information. These differences in F0, therefore, may not only influence how infants respond to the singing environment in the NICU but may also convey biological and kinship-specific cues that can affect neural functioning and development in this sensitive population.

### Study objectives

The F0 of singing and its impact on neonatal brain development remains under-explored, particularly in the context of NICU environments. Current literature lacks detailed insights into how F0 variations, influenced by the biological sex and kinship of the singing source, affect the short-term neurophysiological responses of premature infants. Therefore, this exploratory study is developed as a complementary ‘single-arm’ extension of the randomized controlled trial (RCT; clinicaltrials.gov unique identifier: NCT06398912), aiming to address this research gap by isolating and further examining the neurophysiological mechanisms identified in the original RCT protocol. Specifically, this single-arm study focuses exclusively on the intervention group structure to investigate how different F0 profiles from human voices across various kinship dynamics (i.e., mother, father, male, and female music therapists) influence the brain activity of NICU infants.

The rationale and value for following this presentation approach at this stage (acknowledging of course the inherent limitations of this approach such as the absence of a direct comparison group and weaker causal interpretations) is twofold. First, and most importantly, reporting the single-arm protocol design and its preliminary data alone contributes significantly to the broader field by offering a rigorous methodological template, which can guide future research and intervention designs in similarly sensitive neonatal settings. Second, it allows for a detailed examination of individual and within-subject EEG response patterns to take place, providing nuanced insights that might be obscured by group-level analysis typical in RCTs. Therefore, we believe the current approach is scientifically justified, as it forms a critical foundation for subsequent comprehensive analyses within the context of our broader RCT.

Based on the above, the primary research question of the study reported here is: *Does a structured sound and music-based intervention, delivered by four different facilitators (mother, father, male and female music therapist) as determined by their fundamental frequency (F0) and kinship, result in a differentiated short-term electroencephalographic effect footprint in NICU infants?*

Our research hypotheses are as follows:

- *H1*: We hypothesize that when premature newborns are exposed to a live singing intervention provided by their mother (high fundamental frequency – biological kinship) compared to the father (low fundamental frequency – biological kinship) or an unrelated male/female music therapist, they show statistically more visible short-term positive oscillatory differentiations in their brain activity.

- *H0*: We assume that there is no interaction between the fundamental frequency and the kinship profile (mother, father, man, woman) in the infant’s reactions, and that neither the fundamental frequency nor the human profile has a primary effect.

To answer the research question and study its hypotheses, this study will utilize electroencephalography (EEG) to monitor and analyze the brain activity of NICU infants. By comparing EEG patterns across different music delivery conditions of F0 and kinship source (i.e., music facilitators), the findings are expected to provide valuable insights that can be leveraged to enhance neurodevelopmental outcomes in premature infants.

## Methods

### Study design

**Fig 1:**
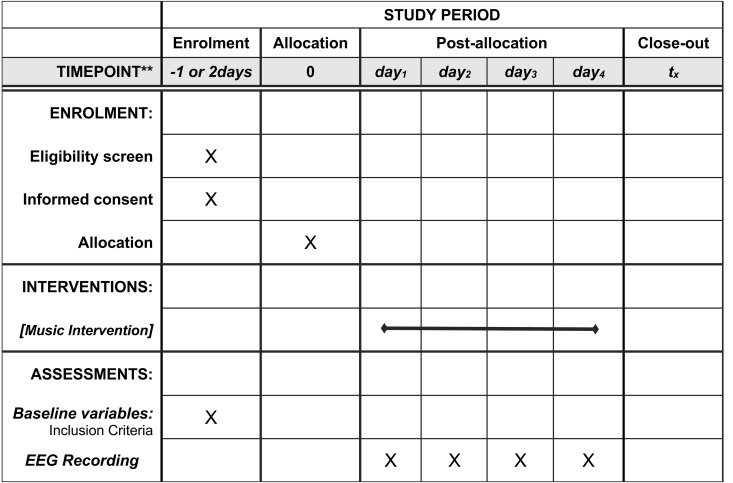
Schedule of enrolment, intervention and assessment.

**Fig 2:**
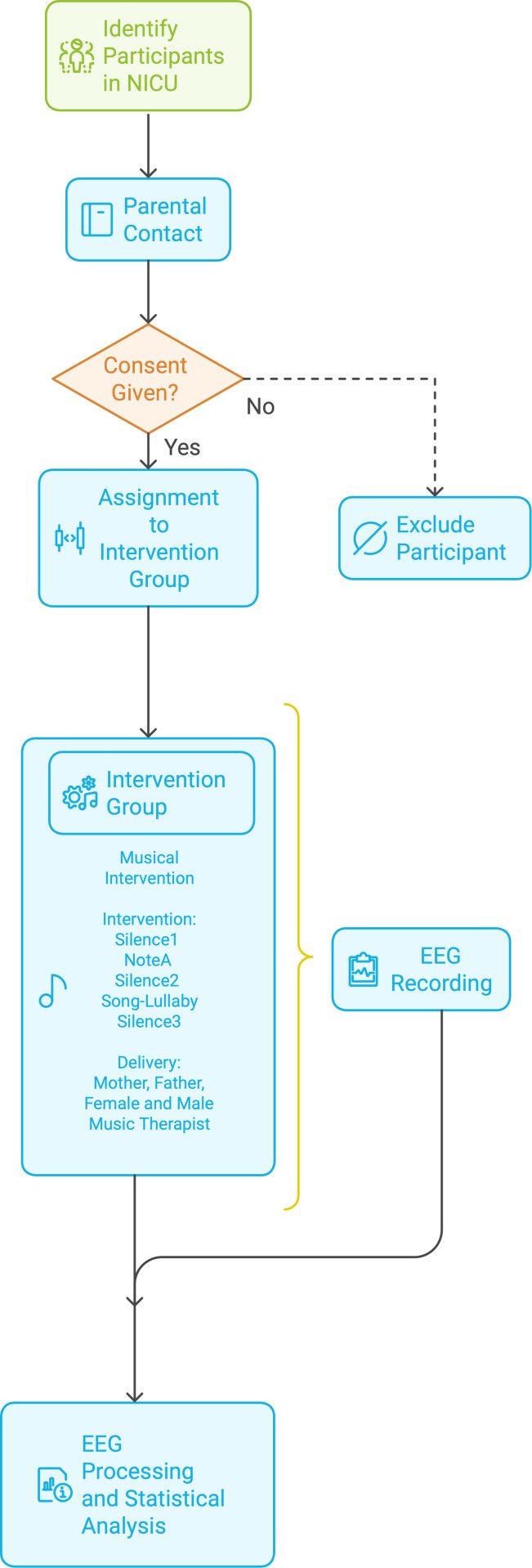
Study Protocol Flow Chart.

Between 06.2023 and 12.2025, all premature newborns who meet the specific inclusion criteria (detailed below) in the NICU of the ‘Panagiotis and Aglaia Kyriakou’ Children’s Hospital in Greece will be screened, and those who qualify will be invited to participate in this exploratory study. During their stay in the NICU, this group of premature infants will be exposed to a structured live singing intervention – including varying investigative conditions of silence and singing – for four days within one week. In terms of measurements, electroencephalography (EEG) will be used to record the newborns’ brain activity throughout their participation in the exploratory study, while video data will be also recorded, forming an auxiliary part to the EEG data collection process. Blinding of the intervention is difficult to implement in this research context due to the direct involvement of parents and facilitators in delivering the singing intervention, which makes it challenging to conceal the nature of the intervention from those administering or receiving it. Based on all the above, the schedule of enrollment, intervention and assessment can be seen in Fig 1 while a flowchart of the study design is shown in Fig 2.

In this design, although the EEG will be the primary tool of data collection and analysis, the rationale for incorporating video data is multifaceted. Firstly, the video recordings will complement the electroencephalogram (EEG) recordings in offering a comprehensive parallel view of the infants’ neural and behavioral responses. Furthermore, the videos will serve as a tool for quality control by ensuring that the prescribed research protocol is uniformly followed by all facilitators, contributing to the scientific integrity of the research. Finally, the videos will allow for a later examination of any synchrony or coordinated interactions between the infant and the facilitator, shedding light on phenotypical – not able to be measured by the EEG – aspects of the intervention.

The live singing intervention will be delivered by four different profiles of individuals-facilitators: a male music therapist, a female music therapist, the mother, and the father of the newborn. During the delivery of the intervention (explained in more detail below), there will be five ‘conditions’ measured: Silence1 (180 seconds), Note A (60 seconds), Silence2 (180 seconds), Song (90 seconds) and Silence3 (180 seconds).

It is important to note that the study protocol will be conducted in a dedicated room, largely isolated from the ambient noise typically found in the rest of the NICU. This aspect of the study design is crucial for two main reasons:

Firstly, it will ensure the comfort and safety of the participating newborns. All actions taken in the context of the NICU require adherence to the recommended standards for the NICU sound environment. Therefore, the importance of a well-managed acoustic environment in reducing stress and discomfort for our participating infants is of paramount importance. Such a consideration has been consistently highlighted both for clinical and research settings alike [32] and a sound-controlled room within the NICU seems to be the only viable option for achieving the necessary level of sound management for our participants, without disrupting other clinical operations. More specifically, in the NICU context, when considering the operational sound as outlined by White, Smith, Shepley, et al. [31], the combination of continuous background sound and operational sound should not exceed an hourly L_eq_ of 45 dB and an hourly L_10_ of 50 dB, both A-weighted slow response, with transient sounds or L_max_ not exceeding 65 dB. Our study, with sound exposure lasting less than 3 minutes per hour, will strictly adhere to these guidelines. However, the transient nature of singing, distinct from the continuous and better-controlled sound pressure levels typically found in continuous speech, might lead to brief instances where we slightly exceed these thresholds by about 15 dB. According to evidence, even professional singers, and more so untrained individuals like the parents participating in our study, are not able to produce transient peak Sound Pressure Levels (SPLs) below 78–79 dB when singing softly [70]. However, it has been noted that in environments with lower ambient noise, humans can more easily maintain a soft vocal output because the reduced background sound allows for greater control and minimizes the need to compensate with higher volume [71]. Therefore, we believe that a dedicated room will significantly aid in ensuring the correct application and viability of the aforementioned recommendations – by providing an acoustically controlled setting that minimizes ambient noise and facilitates softer singing performance – as compared to the open NICU floor, thus preventing the intervention from inadvertently exceeding the recommended soundscape limits.

**Fig 3:**
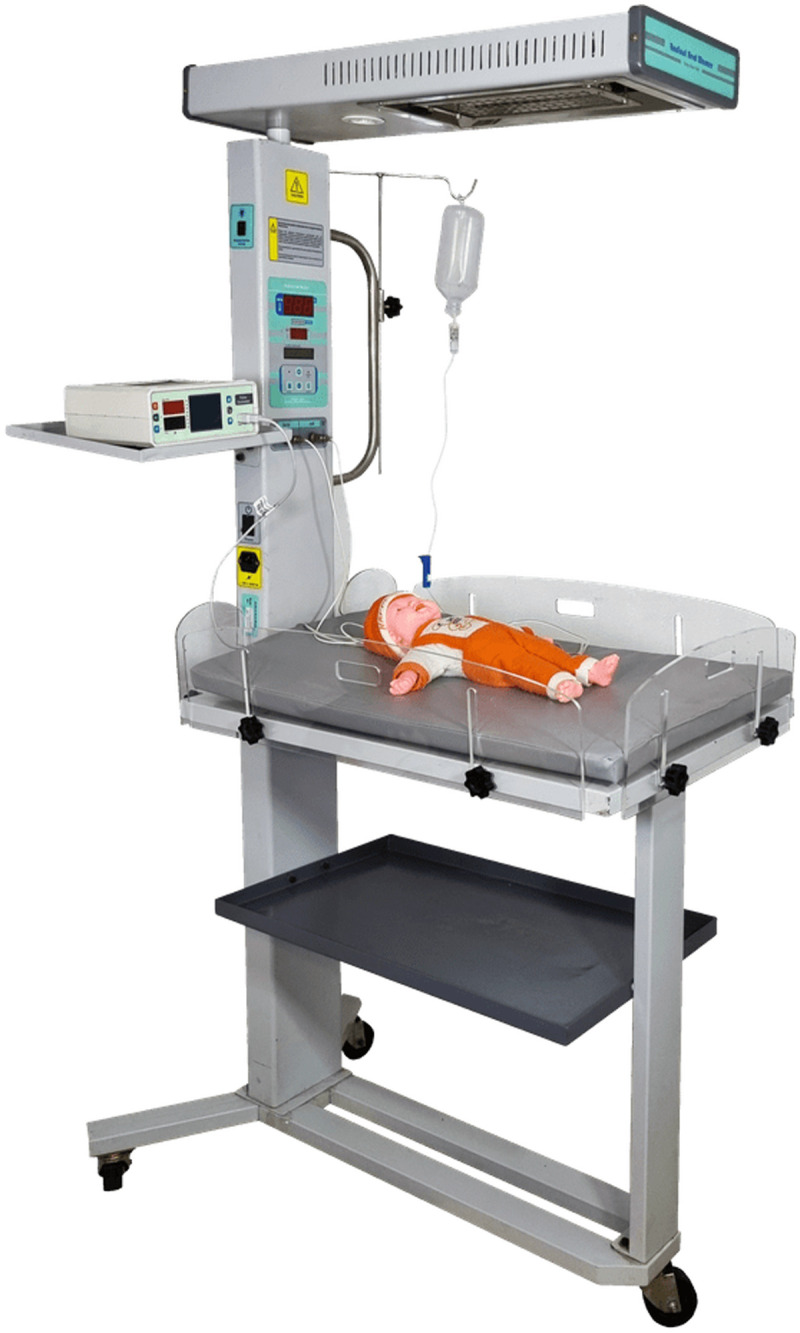
Open cot – Radiant warmer.

Secondly, recent research, including a study by Restin et al. [72], indicates that incubators can be significant sources of noise, especially in the open environment of the NICU floor. To mitigate this issue, we plan to use an open cot/radiant warmer (as depicted in Fig 3) in the dedicated room when applicable, or alternatively, bring the entire incubator into the room. This approach will help minimize or eliminate noise from the incubator that might reach the infant. As an additional precaution to all the above, a dB measuring device will be also stationed in the dedicated room, outside the incubator where applicable, to monitor noise levels throughout the intervention.

### EEG tool, data collection and processing

EEG is an electrophysiological imaging tool commonly used in medicine and research. The electroencephalogram measures changes in electrical dynamics resulting from the formation of electrical dipoles during nerve stimulation. The EEG signal consists of several brain waves that reflect electrical activity in the brain, based on electrode placement and function in adjacent brain areas. EEG-based changes in response to interventions have become a popular method for investigating brain function in patients with various brain and mental health disorders (e.g., [73]), as well as in healthy individuals (e.g., [74]). Moreover, EEG techniques have been successfully applied for many years in both premature newborns and full-term infants (e.g., [75–76]).

In our study, we decided to use the **nëo™** EEG monitoring unit by ANT, a specialized tool designed for both routine and advanced neonatal care in NICUs. Eight high-precision channels found on a clinical-grade infant-sized cap are applied on our participants, connected to a research-grade amplifier with an input impedance exceeding 1 GΩ and advanced noise reduction. We chose this EEG system as it supports neuroprotective intervention screening, offering a maximum sampling rate of 512 Hz and 24-bit resolution.

For the raw EEG data preprocessing part, we have configured a pipeline specialized for the premature infants (as shown in supplementary S1 Data, and presented in [77–79]), while for the processing part we followed and adapted to our protocol a specialized for the premature infants’ population quantitative EEG (qEEG) software developed for neonatal EEG by O’Toole & Boylan [80]. This software has been selected as our preferred EEG processing tool due to its highly informative, detailed, and objective output. Coupled with our preprocessing pipeline, this tool proved to be particularly advantageous for our research, as it allowed for the analysis of various EEG features such as spectral power, coherence, and connectivity among EEG channels, taking into consideration the intervention’s musical conditions and their short delivery profile.

We aimed for this comprehensive EEG setup to enhance our ability to interpret the complex EEG data from our participants in greater detail, as these infants, compared to term infants, toddlers, and adults, exhibit unstable physiological and electroencephalographic states – such as signal continuity/discontinuity [81,82] and increased artifact generation [83–84] – making it significantly more challenging to identify patterns and trends in the EEG data collected following our musical protocol.

### Participants

Premature newborns who meet the inclusion criteria outlined below are eligible for participation in our exploratory study. These criteria have been carefully selected to ensure that the infants included have the appropriate health profile for safe participation and that the study results will be as accurate and relevant as possible. More specifically, to be eligible, newborns must have been born at 32 weeks of gestation or less and weigh at least 1000 grams, while not exceeding 38 weeks of postmenstrual age (PMA) during the intervention and measurement period. It is essential that potential participants do not have auditory insufficiency in the 8th cranial nerve or excessive pathology in the brainstem, while they must also have negative bilateral ‘transient evoked otoacoustic emissions’ – both verified during the postnatal screening by the in-house audiologist.

The eligible participants must also be in stable health, with no imminent risk of death, and must not be sedated or require sedative drug administration during the study, since this will introduce confounding factors related to brain activity that could subsequently alter the study’s findings. Any congenital, genetic, or chromosomal abnormalities, neonatal sepsis, or nuclear jaundice will also lead to exclusion, as these conditions could potentially influence brain function and interfere with the study’s results. Additionally, the participants must not be exposed to maternal use of illicit drugs during pregnancy, while mechanical ventilation used for their life support – that generates excessive noise, such as high-frequency oscillatory ventilation – is another exclusion criterion.

Should a participant be diagnosed with a disease or dysfunction during the course of the study, they will be automatically excluded if the medical staff recommends their removal for health reasons. Additionally, the study protocol mandates that no external musical stimulation, including singing, be allowed for the enrolled participants during the admission period. Non-compliance with this guideline will result in the exclusion of the neonate from the study to maintain the integrity of the experimental conditions and data collection process.

### Power calculation and sample size

In situations where an exact effect size cannot be derived from prior research or pilot data, as in our case, using the Minimum Effect Size of Interest (MESI) [85] is advantageous for guiding sample size estimation. The MESI focuses on the smallest effect size that is considered practically or clinically significant within the specific context of a study, ensuring that the power analysis is calibrated to detect effects that are both meaningful and impactful.

More specifically, for our single-arm exploratory study, the MESI was set at a small-to-medium (conservative) effect size of 0.35. Several methodological reviews support the use of such conservative thresholds to ensure that only differences with true clinical impact are detected. For example, Althubaiti [86] outlines that when using conventional effect size benchmarks – commonly adopted in regression power analyses – the effect (f^2^ = 0.35) is selected to focus on robust differences that justify the exposure of vulnerable populations to research procedures. In addition, Kang [87] demonstrates how G*Power incorporates these standardized benchmarks into its sample size calculations, emphasizing that using such conventional values ensures that the study is powered to detect only those effects that are both statistically significant and clinically meaningful. Even though our design is based on a repeated measures ANOVA rather than regression analysis, the principle remains: a higher MESI minimizes the risk of detecting trivial effects, which is particularly important given the ethical and practical constraints in neonatal research.

Most importantly, from a clinical perspective, neonatal studies must be designed to target effects that will lead to meaningful changes in patient care. Reviews in neonatal contexts (e.g., [88]) stress that interventions in this vulnerable population should only be implemented if they can yield substantial improvements in clinical outcomes, while ethical considerations – such as minimizing exposure to potentially stressful interventions – further support the choice of a conservative MESI. Particularly, Akoglu [89] emphasizes that effect size selection should reflect the minimal clinically important difference. In result, after reviewing the relevant clinical and methodological literature and consulting with field experts involved in our research protocol, the aforementioned MESI threshold was deemed appropriate, ensuring that our power analysis is finely tuned to detect only those effects that hold substantial clinical significance, thereby safeguarding patient care by focusing on differences that are both statistically robust and practically meaningful.

With our designated MESI – as explained above – and by setting alpha at 0.05 and power at 0.80, we conducted an a priori power analysis using G*Power 3.0 for an ANOVA: Repeated measures, within factors design. This analysis incorporated our 5 repeated measurement conditions (Silence1, NoteA, Silence2, Song, Silence3) to account for the experimental structure of the study, with a correlation among repeated measures and a non-sphericity correction to reflect realistic assumptions regarding the correlations and potential sphericity violations among repeated measures.

The power analysis indicated that 14 participants would be sufficient to achieve the desired power level of 80% to detect the specified effect size, with an actual power of 83% (Table 1). We should clarify that in this case we chose G*Power 3.0 as this is more tailored to adjustments specific to repeated-measures designs, such as accounting for non-sphericity and correlations among repeated measures.

**Table 1:** Power analysis table.

Effect Size Specification	G*Power 3.0 – F tests – ANOVA: Repeated measures, within factors	
**Tool**	G*Power 3.1.9.7	
		
**Analysis:**	A priori: Compute required sample size	
**Input:**	Effect size f(V)	0.35
	α err prob	0.05
	Power (1-β err prob)	0.80
	Number of groups	1
	Number of measurements	5
	Corr among rep measures	0.5
	Nonsphericity correction ε	0.75
**Output:**	Noncentrality parameter λ	12.8625
	Critical F	2.845
	Numerator df	3.00
	Denominator df	39.00
	**Total sample size**	**14**
	Actual power	0.83

### Intervention

Preterm newborns who meet the inclusion criteria will be exposed to a specific, in terms of content and exposure timing, live singing intervention four times in a calendar week – once per day. The decision to implement the intervention four times per calendar week was informed by a critical framework evaluation of clinical and practical constraints outlined in Dimitropoulos [90]. According to this evaluation, conducting the intervention more frequently (e.g., 5 times/week or more) could induce excessive stress on infants and caregivers, raising ethical concerns and logistical challenges for maintaining caregiver involvement within the hospital setting beyond one week. Conversely, a lower frequency (e.g., 3 times/week or fewer) is not feasible due to the study’s design, which explicitly involves four distinct facilitator profiles (mother, father, male music therapist, female music therapist). In addition, by conducting the intervention in the proposed way, we expect to observe and record the immediate and short-term neurophysiological effects of the musical stimuli on the infants’ brains, while ensuring that the data reflects the true impact of the intervention, free from external variables that might arise from a more prolonged or sporadic schedule. Furthermore, the specific exposure over this consistent period will facilitate a comprehensive understanding of any cumulative or evolving effects of the intervention across consecutive sessions, allowing for a detailed analysis of the EEG baselines recorded each one of the four times the intervention is delivered.

Most importantly, we aim for the experimental intervention to be always conducted under specific conditions to ensure consistency and optimize the infants’ response to the musical stimuli. More specifically, the intervention will be delivered post-feeding, at least 30 minutes but not more than 60 minutes after. We believe for this timing to be crucial to ensure that the infants are neither too full nor too hungry, both of which could cause discomfort and impact the infant’s ability to remain calm and attentive during the intervention. Feeding influences the infants’ physiological state significantly [91], and selecting this window helps to maintain a stable state, reducing the likelihood of confounding factors related to digestion or hunger that might affect neural responses. Indeed, previous research supports the importance of carefully timing interventions to coincide with naturally occurring alert behavioral states in preterm infants [92,93]. In this context, to ensure optimal attentiveness, infant behavioral states are closely monitored prior to each session, and interventions are initiated only when infants exhibited a quiet alert state at the minimum, defined by lead physicians and nurses as having open eyes, minimal motor movements, and clear responsiveness to gentle external stimuli.

In addition, any other caregiving activities, including kangaroo care (skin-to-skin contact), will be minimized (following the lead physician’s directions) for at least 15–20 minutes prior to the intervention, with the exception of necessary medical care. This precautionary approach aims to minimize potential satellite processes associated with caregiving that could inadvertently lead to overstimulation or physiological stress. Specifically, these concerns include inadequate thermoregulation potentially causing overheating or chilling, compromised respiratory function due to positioning, accidental dislodgment of critical medical devices, mild fluctuations in heart rate or blood pressure, and caregiver anxiety or suboptimal handling, all of which may impact arousal levels and influence the basal neural activity recorded by EEG [94,95]. By ensuring a period of relative rest before the intervention, we aim to create a consistent starting point for all participants, thereby improving the accuracy and reliability of the neurophysiological data collected. Finally, it should be also noted that the facilitators’ order of intervention delivery for each infant will be changed between participants to achieve better randomization and to avoid potential confounding variables due to a set order of intervention delivery.

As far as the live singing intervention’s structure is concerned, this will take place in two main singing stages complemented by three distinctive blocks of silence, before, between and after each sung session as shown in the following list.

* 180 seconds of silence (*Silence1; Baseline1*)
- 1st singing stage: Signing the specific Note A, repeated *ad libitum* for a total of 60 seconds. (*NoteA*)
* 180 seconds of silence (*Silence2; Baseline2*)
- 2nd singing stage: Singing an original song (please see S2) suitable for the NICU environment in terms of structure, acoustic information composition, and volume level (dB), with a total duration of 90 seconds. (*Song*)
* 180 seconds of silence (*Silence3; Baseline3*)

We decided to include the two different musical stimuli – the note A and the lullaby song – in order to differentiate between the effects of a simple, consistent auditory stimulus and a more complex, emotionally rich stimulus. Note A represents a controlled and repetitive sound, allowing us to observe basic auditory processing in the infants’ brains. The lullaby, on the other hand, is a complex acoustic stimulus designed to mimic a natural caregiving experience, thereby engaging additional cognitive and affective neural processes. By comparing these two types of stimuli, we aim to understand how different levels of auditory complexity impact neurodevelopmental outcomes.

We furthermore decided to introduce the periods of silence before and after each singing stage to facilitate a return to the baseline of brain activity between the two singing stages, as well as to reduce the potential impact of any residual neural activity from the previous stage. This approach will enable us to achieve a more accurate assessment of the effects of each individual singing stage, and it will also allow for a clearer comparison of the developmental trajectory of the EEG baselines. By incorporating these controlled intervals of silence, we aim to isolate the neurophysiological effects specific to each condition, thus providing a more precise understanding of how the infants’ brain activity evolves over the course of the intervention conditions as well as sessions.

In regard to the singing stages, as stated above, all facilitators will be asked to sing at the same distant location from the cot, maintaining in this way consistent spatial sound parameters of stimulus delivery. For the first stage of the singing part (NoteA), each facilitator will be given a tuning fork (a forked acoustic component that produces sound upon impact and quickly silences) tuned to the musical note A (440 Hz) and asked to sing based on its produced sound the musical note A as best as they can. All facilitators will need to repeat the specific note *ad libitum* at any possible and convenient to them frequency that corresponds to this specific note (i.e., A2: 110 Hz; A3: 220 Hz; A4:440 Hz; A5:880 Hz) over 60 seconds at this stage. The tuning fork has been chosen to be used as it is a safe acoustic accessory for accurately reproducing the tone the facilitator needs to hear as an acoustical template when inserting it into their ear, ensuring at the same time that the infant will not be able to hear it and be affected by it.

For the second singing stage (Song), each facilitator will need to sing *a cappella* a complex acoustic stimulus with organized sounds in the form of a lullaby, specifically created and composed for the study by a professional musician and music therapist (a member of the research team). This lullaby was aimed to have a unique structure and acoustic information composition adhering to the known scientific evidence related to infants’ sound and music perception capabilities [96,97]. The song lasts 90 seconds and features a soft melodic line, gentle rhythm, simple harmonies in a minor key, and soft vocal timbre, influenced by folk characteristics found in Greek lullabies. Its tempo is expected to be approximately 60–75 beats per minute, following the rhythm of the seconds hand of the clock found on site as best as possible for consistency of delivery.

Finally, to ensure the best possible implementation of the intervention – particularly regarding the singing process – all parents of participants who meet the study’s inclusion criteria will receive 30 minutes of training before the actual intervention. This training will be conducted by a member of the research team who is a professional musician, singer, and music therapist. This pre-intervention session aims to equip parents with the necessary skills and understanding to enhance the intervention’s effectiveness, ensuring close consistency among the different facilitators. Additionally, the training session is designed to provide a calming influence on the parents, which can reduce stress and anxiety for both the infants and their caregivers during the delivery of the intervention. We believe that this pre-intervention training will empower parents as active participants in their child’s care, improving adherence and contributing to a more supportive and therapeutic environment throughout our study in the NICU.

### Protocol amendments

The study protocol reported here is the second configured version, following several adjustments made for both practical and scientific purposes. An evaluation process was carried out in line with established medical research guidelines [98] and the findings informed a series of recommendations, which were incorporated into the current study design. A detailed account of the evaluation process as well as the changes made from the original protocol are available in Dimitropoulos [90] in full, and briefly in S3.

### Outcomes and statistical analysis

To rigorously evaluate the effects of our live singing intervention, we decided to employ a series of advanced statistical methods tailored to the specificities of the study design and data characteristics. The complete analysis focused on the EEG-based outcome measures, with an emphasis on Spectral Power (primary outcome) and Spectral Entropy (secondary outcome), across the Delta frequency band.

Special attention was given to the Delta frequency band as it is widely recognized for its relevance in neonatal brain activity [99–100]. The Delta frequency band, typically ranging from 0.5 to 4 Hz, is the slowest of the EEG frequency bands and is a critical marker of brain maturation. Higher absolute power in the delta frequency band has been associated with more favorable long-term neurodevelopmental outcomes, reflecting the progression of underlying neural processes during early development [99]. Given its prominence in this context, we believe that the Delta frequency band can be an essential marker for assessing the impact of sound and music on the neurophysiological state of our participants, providing in result critical insights into how these infants process auditory stimuli and the extent to which our intervention influences brain activity patterns.

More specifically to the design of our statistical analysis process and given the study’s repeated measures design and the nested data structure (with multiple observations per infant), a mixed-effects model was decided to be employed to analyze first the Delta Spectral Power, allowing for the inclusion of both fixed effects (conditions and facilitators) and random effects (variability across individual infants), while also meticulously fitting the model to account for the inter-individual variability after isolating the impact of the different intervention conditions and facilitator profiles.

Specifically, to the Spectral Power approach, we decided to focus on this continuous measurement EEG feature as it can provide information about the intensity and prevalence of different types of neural oscillations [101] and it can help us better understand how the fluctuating premature infant brain activity changes over time. The thorough statistical analysis approach we propose here on the Delta Spectral Power is expected to generate fixed and random effects estimates, while to further elucidate the specific differences between condition/facilitator combinations, a Least Significant Difference (LSD) post-hoc test will be also conducted on the model’s outcome, aiming to identify which specific pairs of conditions or facilitators exhibit significant differences in brain activity, despite the increased risk of Type I errors due to multiple comparisons.

Parallel to the analysis of Delta Spectral Power, a similar mixed-effects model will be applied to the Delta Spectral Entropy data collected, following the same consideration for random and fixed effects, and aiming to explore how variations in spectral entropy, influenced by the different intervention profiles, reflect the neurophysiological responses of the infants. We decided to further study the EEG feature of Spectral Entropy in this context as it can offer a different dimension of analysis compared to Spectral Power and reflect in more detail the overall organization and complexity of the oscillations studied through the Delta Spectra Power approach. More specifically, Spectral Entropy is considered a measure of the complexity or irregularity of a signal’s frequency distribution [102], quantifying the degree of disorder or unpredictability within the power spectrum of a specific signal – in our case the Delta frequency band. A decrease in Spectral Entropy could indicate a transition to more synchronized and less complex neural activity, while conversely, higher Spectral Entropy might suggest more distributed and diverse neural activity, which could be linked to alertness or cognitive engagement.

Infusing this extra step of statistical analysis on our collected EEG data may be proved particularly valuable because it may reveal subtle changes in brain activity that may not be apparent through power analysis alone. As with the Spectral Power analysis, and subsequent to the mixed-effects model for Delta Spectral Entropy, a Least Significant Difference (LSD) post-hoc test will be also employed to investigate the differences between condition/facilitator combinations as in the previous approach.

### Ethics

This study has been meticulously designed with a strong commitment to ethical standards, ensuring the protection and well-being of all participants. The research protocol has been thoroughly reviewed and approved by the ‘Panagiotis and Aglaia Kyriakou’ Hospital’s Scientific Council, under the Institutional Review Board (IRB) with protocol number 11333, 09th/04-05-2023 (Θ: 9). Additionally, the study has been registered on the clinical trials platform (www.clinicaltrials.gov) under the unique identifier NCT06398912, ensuring transparency and adherence to global research standards.

Participation in this study is entirely voluntary, and informed consent is a central component of the ethical process. Therefore, parents of identified premature infants who meet the inclusion criteria are invited to a detailed one-on-one meeting with the research team, in order to receive comprehensive information, both orally and in writing, regarding the study’s objectives, methodologies, and procedures. This meeting covers all aspects of the intervention and data collection, including the non-invasive nature of the research tools, such as music and EEG, while also addresses any concerns related to selection, participation, withdrawal, confidentiality, and the study timeline, ensuring that parents are fully informed before making a decision.

During this meeting, the voluntary nature of participation is emphasized, with parents being assured that their decision to participate or withdraw at any point will not affect their relationship with the researchers or the medical staff at the Panagiotis & Aglaia Kyriakou Children’s Hospital. They are also informed that in the event of withdrawal, all data associated with the participant will be immediately deleted, ensuring no residual information remains.

Considering the sensitivity of the particular context, and to further protect the participants’ privacy and confidentiality, all collected data are de-identified and pseudonymized at the source (i.e., embeded function of the **nëo™** EEG monitoring unit during the extraction process), with each dataset assigned a unique identification number. Personal identifiers, such as names, are not linked to the data in any study analysis, report, or publication phase, while parents are informed of their right to access the data collected from their infants at any time. Once parents have received all relevant information and have had their questions answered, they are asked to provide written consent, indicating their agreement to participate in the study.

At this point, we should mention that despite its non-invasive nature, the study recognizes potential risks, such as the overstimulation of infants due to sound exposure and stress related to handling. To mitigate these risks, sound levels are closely monitored using a dB dosimeter – as already mentioned – and interventions are scheduled in this way so to ensure adequate rest periods for the infants. It has already been pointed above that the study is conducted in a specially designated, quiet room within the NICU to minimize stress, while moreover, the application of EEG electrodes, a key part of the study, is performed by specialists trained in neonatal care, further reducing the risk of discomfort or stress for the infants. In case of any unforeseen medical complications or adverse reactions, the study protocol includes provisions for the immediate cessation of the intervention and prompt medical evaluation, ensuring the safety of the participants.

### Trial status

#### Participants.

Since the ethical approval by the IRB and the Hospital Scientific Board in May 2023 and until September 2024, 11 participants have been measured in this single-arm exploratory study. However, data from only five participants from the experimental group have been analyzed so far, comprising the preliminary data reported at this stage. This small preliminary analysis cohort is consisted by two female and three male premature infants ranging between 28 and 32 weeks of gestation (i.e., specifically 199–227 days of gestation; SD = 12.42 days; Median (75^th^ Percentile) = 227 days). All five participants were measured in their 35th week PMA.

Prematurity in this dataset is underscored by the diverse maternal histories, including one mother who underwent in vitro fertilization (IVF) resulting in dichorionic diamniotic (DCDA) twin pregnancy (described as uneventful); a mother with an IgA nephropathy and a kidney transplantation, coupled with diabetes during pregnancy; one case involving increased resistance in the uterine arteries, raising concerns about potential intrauterine growth restriction (IUGR) and preeclampsia, and one last case experiencing a pregnancy complicated by IVF, gestational diabetes, polyhydramnios, and abnormal Doppler findings. Regarding the infants’ treatment, antibiotics and caffeine were commonly administered (typical neonatal care practices employed), with three cases receiving a standard combination of antibiotics and caffeine, while one case specifically involved ampicillin and gentamicin alongside caffeine. In one instance, no medication was reported, indicating a lack of need for such intervention.

#### Results – descriptive statistics.

In terms of the EEG analysis results for this dataset, the descriptive statistics for spectral power in the Delta frequency across different facilitators and conditions are detailed in Table 2 below. The highest mean spectral power was observed during maternal interventions, particularly in the ‘NoteA’ condition (Mean = 1381.3 *μ*V^2^, SD = 1794.5 *μ*V^2^), emphasizing the notable influence of maternal presence in general.

**Table 2:** Descriptive statistics for spectral power on delta frequency (in *μ*V^2^ for Mean and SD).

Facilitator	Condition	Mean	Standard Deviation	Coefficient of Variation (%)
Elena	NoteA	276.1	269.9	97.7
Father	NoteA	657.1	937.4	142.7
Kyriakos	NoteA	273.6	129.5	47.3
Mother	NoteA	1381.3	1794.5	129.9
Total	NoteA	647.1	1047.2	161.8
Elena	Silence1	92.7	83.4	90
Father	Silence1	161.2	175.8	109.1
Kyriakos	Silence1	339	308.7	91.1
Mother	Silence1	703.1	745.9	106.1
Total	Silence1	324	451.8	139.4
Elena	Silence2	114.8	162.3	141.4
Father	Silence2	148.7	103.3	69.4
Kyriakos	Silence2	225.7	137.3	60.9
Mother	Silence2	218	168.8	77.4
Total	Silence2	176.8	141.6	80.1
Elena	Silence3	94.3	84.6	89.7
Father	Silence3	427.6	785.9	183.8
Kyriakos	Silence3	378.3	538.1	142.2
Mother	Silence3	1020.7	1797.1	176.1
Total	Silence3	480.2	995.9	207.4
Elena	Song	79.1	76.2	96.4
Father	Song	574.2	920.8	160.4
Kyriakos	Song	270.2	231.8	85.8
Mother	Song	213.4	185.7	87
Total	Song	284.2	482.5	169.8

#### Statistical Assumptions Check.

**Fig 4:**
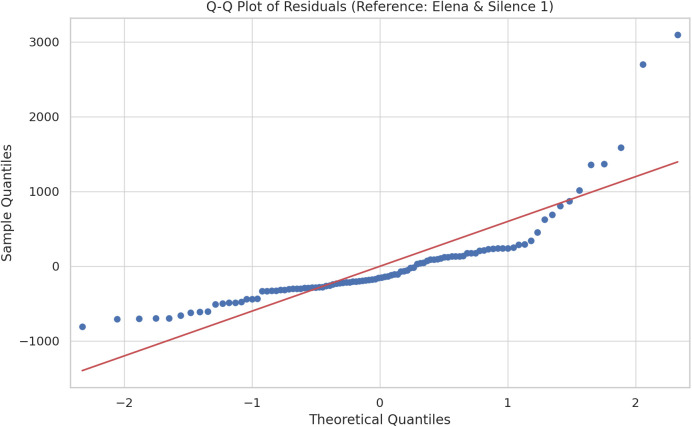
Q-Q Plot of Residuals with a reference point to the female music therapist for the facilitators and the ‘Silence1’ condition for the five intervention conditions.

Following the descriptive statistics, and before running the mixed-effects model as planned, assumptions checks were performed by creating a Q-Q plot to assess the normality of residuals and conducting a Breusch-Pagan test for homoscedasticity. The Q-Q plot (Fig 4) indicated that residuals followed an approximately normal distribution, although this approximation was influenced by the presence of a few outliers. Due to the limited sample size available, we chose in this case not to exclude these outliers from the analysis to avoid further reducing the already limited statistical power. Practically, retaining these outliers means our estimates and associated inferences remain conservative, reflecting real-world variability more accurately but potentially inflating error variance slightly. Additionally, the Breusch-Pagan test yielded a p-value of 0.066, suggesting that residual variance remained sufficiently consistent across the fixed effects, thus indicating that the assumption of homoscedasticity holds.

### Outcomes measures

#### Primary outcomes.

**Fig 5:**
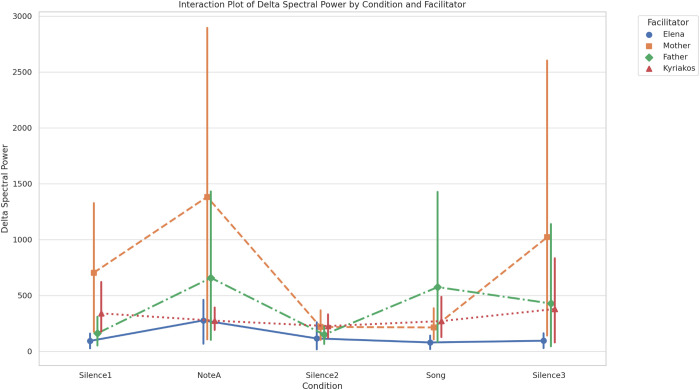
Interaction Plot of Delta Spectral Power by Condition and Facilitator. This figure illustrates the mean delta spectral power (y-axis) across five experimental conditions – Silence1, NoteA, Silence2, Song, and Silence3 (x-axis) – for the four facilitators (Elena, Mother, Father, and Kyriakos). Error bars represent the standard deviation (SD) of individual measurements, highlighting the variability of infants’ responses within each facilitator’s group.

Considering the model assumptions to hold, and given the continuous nature of our measures, we run first a linear mixed-effects model on the Spectral Power of the Delta frequency as decided, which included 100 observations across 5 groups, with each group comprising 20 observations at multiple time points. The model converged successfully with a scale parameter of 404,270.9768 and a log-likelihood of −739.3134. The coefficients for each condition and facilitator, along with their standard errors and p-values, are reported in full in Table 3. The model revealed a significant effect of the ‘facilitator’ parameter on spectral power [F(3,88) = 3.627, *p* = 0.016}, while the ‘condition’ parameter did not show a statistically significant sole effect overall [F(4,88) = 1.669, *p* = 0.164)]. On the contrary, when we combined the effects across different conditions and facilitators (please see interaction plot in Fig 5), the model revealed that although the Delta Spectral Power generally fluctuates, some interesting findings can be drawn based on this synthesis.

**Table 3:** Coefficients for each condition and facilitator with SE and *p* values.

Condition/Facilitator	Coefficient (Mean)	Standard Error	p-value
Elena	131.442	181.968	.148
Father	393.818	181.968	.593
Kyriakos	297.398	181.968	.025
Mother	707.362	181.968	.002
NoteA	647.105	192.757	.112
Silence1	324.049	192.757	.466
Silence2	176.847	192.757	.022
Silence3	480.281	192.757	.135
Song	284.243	192.757	.075

More specifically, we realized that mostly during the ‘NoteA’ condition (as also observed in most of the other conditions), there was a significant increase in Delta Spectral Power for the ‘mother’ facilitator, while surprisingly, the ‘Song’ condition showcased the highest impact particularly for the ‘father’ facilitator. As far as the ‘female music therapist (Elena)’ facilitator is concerned, the most stable and lowest Delta Spectral Power across all conditions was observed, indicating a consistent but minimal impact, while the ‘male music therapist (Kyriakos)’ facilitator showed moderate variability with minor peaks during the ‘NoteA’ and ‘Song’ conditions. Please note here, that especially for the ‘Song’ condition, the ‘male music therapist (Kyriakos)’ facilitator ranked second in impact after the ‘father’ facilitator. To further clarify these specific differences, post-hoc comparisons using the Least Significant Difference (LSD) test were conducted as previously decided, revealing that Delta brain activity was significantly higher with the ‘Mother’ facilitator compared to ‘Elena’ (M = 575.92, *p* = 0.002), ‘Kyriakos’ (M = 409.96, *p* = 0.025), and ‘Father’ (M = 313.55, *p* = 0.085) – although for this latter coming quite close to being significant. Additionally, the ‘NoteA’ condition elicited significantly higher brain activity than the ‘Silence2’ condition (M = 470.26, *p* = 0.022) and showed close trends towards significance when compared to the ‘Silence1’ (M = 323.06, *p* = 0.112) and ‘Song’ conditions (M = 362.86, *p* = 0.075).

#### Secondary Outcomes.

**Fig 6:**
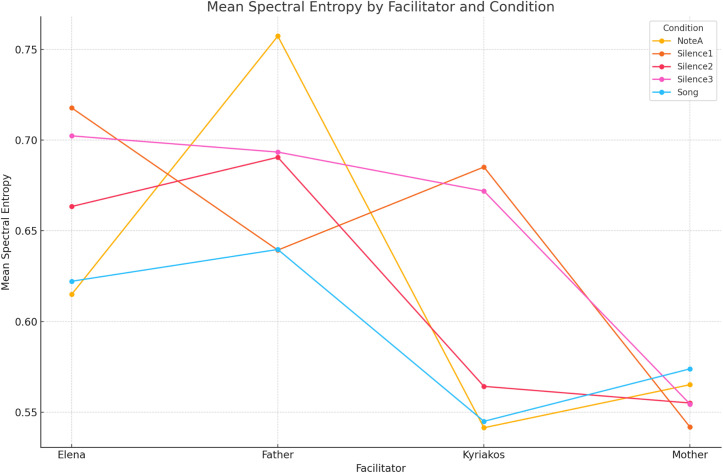
Mean spectral entropy within the Delta band, showing a significant effect of the ‘facilitator’. The ‘father’ shows the highest mean entropy and the ‘mother’ the lowest.

Finally, as already mentioned, in addition to the analysis of Delta spectral power, we also explored the spectral entropy within the Delta frequency band to assess the complexity and predictability of the EEG signals under different facilitators and conditions. The mixed-effects model analysis for spectral entropy revealed once more a significant effect of the ‘facilitator’ parameter on entropy values [F(3, 88) = 2.744, *p* = 0.048], while the spectral entropy across the ‘conditions’ parameter did not exhibit a significant overall effect [F(4, 88) = 0.380, *p* = 0.822]. Especially for the former result, underscoring that the facilitator’s role notably influences the variability and regularity of the EEG signals, we realized that, specifically, the ‘father’ facilitator was associated with the highest mean entropy (M = 0.684, SE = 0.133), while the ‘mother’ facilitator showed the lowest mean entropy (M = 0.558, SE = 0.133) (please see also Fig 6); although pairwise comparisons did not reach statistical significance (in all cases except the close to significance trend of the ‘father’ vs ‘mother’ comparison) after Bonferroni correction (please see Table 4).

**Table 4:** Pair-wise comparison of the ‘facilitator’ condition after Bonferroni correction.

Comparison	Mean Difference	Standard Error (SE)	p-value
Elena vs. Father	−0.02	0.049	1
Elena vs. Kyriakos	0.063	0.049	1
Elena vs. Mother	0.106	0.049	0.207
Father vs. Kyriakos	0.083	0.049	0.589
Father vs. Mother	0.126	0.049	0.075
Kyriakos vs. Mother	0.043	0.049	1

## Discussion

Although there is emerging evidence that musical interventions can positively influence the neurodevelopment of premature infants, existing studies often suffer from limited control over confounding variables, and a lack of rigorous neuroimaging-based evidence to support the precise neural mechanisms underlying these effects. Against this backdrop, the aim of the present study is to fill these gaps by providing robust data on the effects of a structured singing intervention, specifically focusing on the F0 of the voice and kinship of the music facilitators parameters, on the brain activity of premature infants in a NICU setting. Building upon this foundation, some of the key strengths of the protocol presented here include: (1) the adoption of a single-arm exploratory design that enables detailed within-subject and between-session comparisons; (2) the employment of an advanced neuroimaging technique to precisely monitor and study neural correlates of the intervention; and (3) the consideration of a variety of caregivers – in terms of kinship and voice register – to deeper explore the specific impact of singing on premature infant brain development.

More specifically, the protocol was meticulously designed to ensure a structured framework, while designed as a single-arm exploratory study, allowing for an in-depth exploration of the intervention’s specific impact through both within-subject and between-session comparisons. This design enables the observation of changes in individual participants over time, providing insights into intra-individual variability and the effects of repeated exposure to the intervention. Additionally, between-session analyses will offer a broader understanding of how the intervention might generate consistent patterns across participants, thereby identifying key mechanisms of action. These analyses are crucial for detecting subtle, short-term neurophysiological responses that might be overlooked in larger controlled trials where individual variability can be masked. Thus, the single-arm design is particularly valuable for revealing complex intervention-outcome relationships and generating hypotheses that inform more targeted and rigorous future research. By focusing on individual responses, the study can highlight nuanced effects, identify key factors influencing brain activity, and ultimately provide foundational data to guide subsequent, more expansive studies.

Furthermore, by employing electroencephalography (EEG) and utilizing a custom-made and adapted EEG (pre)processing pipeline, the effects of our live singing intervention on premature infants can be studied in greater detail. This advanced neuroimaging approach allows for a nuanced understanding of how different auditory stimuli influence brain activity in this vulnerable population. As discussed in previous work [30], neuroimaging-guided interventions should be more extensively introduced in the NICU because they can provide real-time insights into the brain’s response to external stimuli, capturing dynamic changes that might not be observable through behavioral measures alone. In this regard, our protocol not only enhances the precision of our study but also contributes to the development of more tailored applications in the future, optimizing music-based interventions to suit the unique neurodevelopmental needs of each infant in the NICU.

Finally, it is important to point out that the study design we decided to implement includes the chosen variety of caregivers to allow us to provide a more comprehensive insight into the role of emotional and social factors evident and embedded in the treatment process of premature infants in real-world situations and environments. For that matter, on the one hand, the inclusion of both parents in the protocol ensures that the study captures potential sex-specific effects, which could be related to differences in voice register or emotional bonding, while, on the other hand, incorporating professional music therapists as facilitators offers a standardized comparison to the former set of caregivers, helping to differentiate the effects of kinship as well as musical and clinical expertise related to the implementation of music-based interventions in this sensitive context. This diversity in caregivers could not only enrich the study’s findings but may also enhance the generalizability of its results, ultimately leading to more comprehensive recommendations for NICU music-based interventions.

Having said the above, the preliminary findings of our study provide intriguing evidence supporting differential effects of parental voices on neonatal brain activity, particularly highlighting the potential influence of the father’s voice. Specifically, the initial EEG analyses suggest that paternal singing elicited notably higher delta spectral power compared to maternal and therapist singing during the lullaby (Song) condition. This preliminary result aligns well with existing literature [103], which suggests that lower-pitched sounds – typically associated with male voices – may uniquely stimulate neural circuits at this developmental stage due to their distinct bioacoustic properties and the specific auditory needs of fetuses – and by extension premature neonates. Moreover, the father’s voice might confer additional familiarity and emotional significance owing to biological kinship. If further analyses confirm these findings, extended paternal involvement may then offer emotional and social stimuli complementary to maternal care, thereby contributing to more robust caregiver-infant bonding dynamics during this sensitive developmental period.

### Challenges and limitations

Despite the strengths of our study, certain challenges and limitations must also be acknowledged. First, the heterogeneity of the study population, which may potentially include infants with varying degrees of prematurity – as defined by their gestational age – and differing health profiles, may introduce significant variability and complicate the interpretation of the results, as the infants’ responses to the live singing intervention may be influenced by factors beyond the scope of the study. For instance, the inclusion of participants with diverse medical histories, while increasing the generalizability of the findings, also necessitates careful consideration in data analysis and further clinical implementation to account for these differences. Furthermore, external factors, such as the NICU environment, could also influence the study’s conduct and the quality of the data collected. The acoustic environment for once, despite being controlled within the study’s dedicated room, may still introduce variability that could affect the infants’ responses, while the fragile emotional and psychological state of the parents, who are also facilitators in the study, could impact the delivery and reception of the live singing intervention. These factors, although managed to the greatest extent possible, remain potential sources of variability that could influence the outcomes.

Despite these challenges, the potential impact of this study remains highly significant. Its future findings, for once, could be easily integrated into existing neonatal care practices, offering healthcare providers a scientifically grounded method for enhancing the care of premature infants. Furthermore, the broader impact of this study could be substantial as the insights gained from the differential effects of maternal, paternal, and therapist-facilitated singing on neonatal brain activity could inform wider public health strategies aimed at improving neurodevelopmental outcomes for premature infants. Finally, this research may also encourage the expansion of music-based interventions in neonatal care units worldwide, particularly in regions where such practices are not yet widely implemented.

## Conclusion

As this study is still in progress, it is essential to recognize that the findings presented here are preliminary, based on an initial analysis cohort. Therefore, the continuation and completion of the study will be critical for validating these early results and for providing a more comprehensive understanding of the effects of the specific music-based intervention on neonatal brain development. That said, several areas for further investigation have emerged from the process of structuring, fine-tuning, and implementing our study protocol, suggesting for future research to explore the long-term effects of this and other similar interventions, particularly in terms of cognitive and behavioral outcomes as the infants develop. Additionally, more research is needed to understand the specific mechanisms through which other dynamic soundscapes and modes of musical delivery may influence the infants’ brain activity, leading to more targeted and effective interventions. For all the above, it is our hope that the detailed report of our study protocol and its preliminary results provided here, may help future researchers better accommodate their investigation needs and study hypotheses in the emerging field of music-based interventions in the NICU with the help of neuroimaging tools and particularly electroencephalography, leading to new horizons of applicable treatment in this very sensitive yet crucial environment for human development.

## Supporting information

S1 Data Studying newborns’ brain activity in the NICU through a musical intervention: the role of fundamental frequency. (PDF)

S2 Data Human Participants Research Checklist. (PDF)

S3 Data Advancing EEG Processing and Analysis in Neonatal Music Interventions: Introducing a Specialized qEEG Pipeline for Premature Infants in NICU. (PDF)

S4 Data SPIRIT 2013 Checklist: Recommended items to address in a clinical trial protocol and related documents*. (PDF)

## References

[1] Blackburn S. Environmental impact of the NICU on developmental outcomes. J Pediatr Nurs. 1998;13(5):279–89. doi: 10.1016/S0882-5963(98)80013-4 9798363

[2] Bonkowsky JL, deVeber G, Kosofsky BE, Child Neurology Society Research Committee. Pediatric neurology research in the twenty-first century: status, challenges, and future directions post-COVID-19. Pediatr Neurol. 2020;113:2–12. doi: 10.1016/j.pediatrneurol.2020.08.012 32979654 PMC10256134

[3] Santos J, Pearce SE, Stroustrup A. Impact of hospital-based environmental exposures on neurodevelopmental outcomes of preterm infants. Curr Opin Pediatr. 2015;27(2):254–60. doi: 10.1097/MOP.0000000000000190 25635585 PMC4410011

[4] World Health Organization. Preterm birth. https://www.who.int/news-room/fact-sheets/detail/preterm-birth. 2022.

[5] Qattea I, Farghaly MAA, Kattea MO, Abdula N, Mohamed MA, Aly H. Survival of infants born at periviable gestation: The US national database. Lancet Reg Health Am. 2022;14:100330. doi: 10.1016/j.lana.2022.100330 36777383 PMC9903864

[6] Gebreheat G, Teame H. Survival and mortality of preterm neonates in a neonatal intensive care unit in Northern Ethiopia: a retrospective cohort study. Sci Rep. 2022;12(1):600. doi: 10.1038/s41598-021-04521-z 35022458 PMC8755721

[7] Natarajan G, Shankaran S. Short- and long-term outcomes of moderate and late preterm infants. Am J Perinatol. 2016;33(3):305–17. doi: 10.1055/s-0035-1571150 26788789

[8] Jarjour IT. Neurodevelopmental outcome after extreme prematurity: a review of the literature. Pediatr Neurol. 2015;52(2):143–52. doi: 10.1016/j.pediatrneurol.2014.10.027 25497122

[9] Akiyama A, Tsai JD, Wy TE, Kamino D, Hahn C, Go CY, et al. The effect of music and white noise on electroencephalographic (EEG) functional connectivity in neonates in the neonatal intensive care unit. J Child Neurology. 2021;36(1):38–47. 10.1177/0883073820947894 32838628

[10] Zhang S, He C. Effect of the sound of the mother’s heartbeat combined with white noise on heart rate, weight, and sleep in premature infants: a retrospective comparative cohort study. Annals of Palliative Medicine. 2023;12(1):11120–11120. 10.21037/apm-22-1269 36747385

[11] Alemdar DK, Özdemir FK. Effects of covering the eyes versus playing intrauterine sounds on premature infants’ pain and physiological parameters during venipuncture. J Pediatr Nurs. 2017;37:e30–6. doi: 10.1016/j.pedn.2017.06.016 28751136

[12] Iyendo TO. Sound as a supportive design intervention for improving health care experience in the clinical ecosystem: A qualitative study. Complement Ther Clin Pract. 2017;29:58–96. doi: 10.1016/j.ctcp.2017.08.004 29122270

[13] Praskach A. (2009). Nature, daylight and sound: A sensible environment for the families, staff, and patients of neonatal intensive care units (Master’s thesis). University of South Florida. Available from: https://scholarcommons.usf.edu/etd/2154

[14] Almadhoob A, Ohlsson A. Sound reduction management in the neonatal intensive care unit for preterm or very low birth weight infants. Cochrane Database Syst Rev. 2020;1(1):CD010333. doi: 10.1002/14651858.CD010333.pub3 31986231 PMC6989790

[15] Gold C, Erkkilä J, Bonde LO, Trondalen G, Maratos A, Crawford MJ. Music therapy or music medicine? Psychotherapy and psychosomatics. 2011;80(5):304. 21720190 10.1159/000323166

[16] Papatzikis E, Agapaki M, Naveena Selvan R, Hanson-Abromeit D, Gold C, Epstein S, et al. Music medicine and music therapy in pediatric care: A systematic review of passive music listening research applications and findings on infant development and medical practice. medRxiv. 2024;2024–04. 10.1186/s12887-024-05275-z PMC11664842 39710645

[17] Erdei C, Schlesinger K, Pizzi MR, Inder TE. Music therapy in the neonatal intensive care unit: a center’s experience with program development, implementation, and preliminary outcomes. Children (Basel). 2024;11(5):533. doi: 10.3390/children11050533 38790528 PMC11120361

[18] Yue W, Han X, Luo J, Zeng Z, Yang M. Effect of music therapy on preterm infants in neonatal intensive care unit: Systematic review and meta-analysis of randomized controlled trials. J Adv Nurs. 2021;77(2):635–52. doi: 10.1111/jan.14630 33200833

[19] Pölkki T, Korhonen A. The effectiveness of music on pain among preterm infants in the neonatal intensive care unit: a systematic review. JBI Evidence Synthesis. 2012;10(58):4600–9. 10.11124/jbisrir-2012-428 27820525

[20] Caine J. The effects of music on the selected stress behaviors, weight, caloric and formula intake, and length of hospital stay of premature and low birth weight neonates in a newborn intensive care unit. J Music Ther. 1991;28(4):180–92. doi: 10.1093/jmt/28.4.180 10160836

[21] Arnon S, Shapsa A, Forman L, Regev R, Bauer S, Litmanovitz I, et al. Live music is beneficial to preterm infants in the neonatal intensive care unit environment. Birth. 2006;33(2):131–6. doi: 10.1111/j.0730-7659.2006.00090.x 16732778

[22] Loewy J, Stewart K, Dassler A-M, Telsey A, Homel P. The effects of music therapy on vital signs, feeding, and sleep in premature infants. Pediatrics. 2013;131(5):902–18. doi: 10.1542/peds.2012-1367 23589814

[23] Reuter C, Bartha-Doering L, Czedik-Eysenberg I, Maeder M, Bertsch MA, Bibl K, et al. Living in a box: Understanding acoustic parameters in the NICU environment. Front Pediatr. 2023;11:1147226. doi: 10.3389/fped.2023.1147226 37051427 PMC10083238

[24] Brown G. NICU noise and the preterm infant. Neonatal Netw. 2009;28(3):165–73. doi: 10.1891/0730-0832.28.3.165 19451078

[25] Vicencio LA, Ananthanarayana RM, Khudr J, Monson BB. Daily sound level exposure for preterm infants in the neonatal intensive care unit. The Journal of the Acoustical Society of America. 2023;153(3_supplement):A160–A160.

[26] Kvaratskhelia N, Rurua N, Vadachkoria SG. Biomedical and psychosocial determinants of early neurodevelopment after preterm birth. Glob Pediatr Health. 2023;10:2333794X231160366. doi: 10.1177/2333794X231160366 36968456 PMC10037732

[27] Hee Chung E, Chou J, Brown KA. Neurodevelopmental outcomes of preterm infants: a recent literature review. Transl Pediatr. 2020;9(Suppl 1):S3–8. doi: 10.21037/tp.2019.09.10 32206579 PMC7082240

[28] Cook KM, De Asis-Cruz J, Kim J-H, Basu SK, Andescavage N, Murnick J, et al. Experience of early-life pain in premature infants is associated with atypical cerebellar development and later neurodevelopmental deficits. BMC Med. 2023;21(1):435. doi: 10.1186/s12916-023-03141-w 37957651 PMC10644599

[29] Papatzikis E, Agapaki M, Selvan RN, Pandey V, Zeba F. Quality standards and recommendations for research in music and neuroplasticity. Ann N Y Acad Sci. 2023;1520(1):20–33. doi: 10.1111/nyas.14944 36478395

[30] Papatzikis E. Neuroimaging-guided music interventions for infants in NICU. JAMA Pediatr. 2024;178(9):853–4. doi: 10.1001/jamapediatrics.2024.1886 39008320

[31] White RD, Smith JA, Shepley MM, Committee to Establish Recommended Standards for Newborn ICU Design. Recommended standards for newborn ICU design, eighth edition. J Perinatol. 2013;33 Suppl 1:S2–16. doi: 10.1038/jp.2013.10 23536026

[32] EFCNI, Sizun J, Hallberg B, et al. European Standards of Care for Newborn Health: Management of the acoustic environment. European Foundation for the Care of Newborn Infants. 2018. Retrieved from https://newborn-health-standards.org

[33] Hu BH, Guo W, Wang PY, Henderson D, Jiang SC. Intense noise-induced apoptosis in hair cells of guinea pig cochleae. Acta Oto-Laryngologica. 2000;120(1):19–24. 10779180

[34] Kamio T, Watanabe K-I, Okubo K. Acoustic stimulation promotes DNA fragmentation in the Guinea pig cochlea. J Nippon Med Sch. 2012;79(5):349–56. doi: 10.1272/jnms.79.349 23123391

[35] Gerhardt KJ, Abrams RM. Fetal exposures to sound and vibroacoustic stimulation. J Perinatology. 2000;20(1):S21–30. 10.1038/sj.jp.7200446 11190697

[36] Bueno C, Menna-Barreto L. Development of sleep/wake, activity and temperature rhythms in newborns maintained in a neonatal intensive care unit and the impact of feeding schedules. Infant Behav Dev. 2016;44:21–8. doi: 10.1016/j.infbeh.2016.05.004 27261553

[37] Akerlund L, Gramming P. Average loudness level, mean fundamental frequency, and subglottal pressure: comparison between female singers and nonsingers. J Voice. 1994;8(3):263–70. doi: 10.1016/s0892-1997(05)80298-x 7987429

[38] Kuo DZ, McAllister JW, Rossignol L, Turchi RM, Stille CJ. Care coordination for children with medical complexity: whose care is it, anyway? Pediatrics. 2018;141(Suppl 3):S224–32. doi: 10.1542/peds.2017-1284G 29496973

[39] van Dokkum NH, Fagan LJ, Cullen M, Loewy JV. Assessing heartsong as a neonatal music therapy intervention: a qualitative study on personal and professional caregivers’ perspectives. Adv Neonatal Care. 2023;23(3):264–71. doi: 10.1097/ANC.0000000000001068 37075326

[40] Haslbeck FB, Bassler D. Clinical practice protocol of creative music therapy for preterm infants and their parents in the neonatal intensive care unit. J Vis Exp. 2020;(155):10.3791/60412. doi: 10.3791/60412 31984968

[41] Guidry-Jorgensen S, Abroms KI, Staffileno BA. The cognitive and emotional development of premature infants: A review of the literature. Journal of Pediatric Nursing. 2011;26(3):220–7.

[42] Borghini A, Pierrehumbert B, Miljkovitch R, Muller-Nix C, Forcada-Guex M, Ansermet F. Mother’s attachment representations of their premature infant at 6 and 18 months after birth. Infant Ment Health J. 2006;27(5):494–508. doi: 10.1002/imhj.20103 28640398

[43] Vrticka P, Vuilleumier P. Neuroscience of human social interactions and adult attachment style. Frontiers in Human Neuroscience. 2012;6:212. 22822396 10.3389/fnhum.2012.00212 PMC3398354

[44] Shoemark H, Hanson-Abromeit D, Stewart L. Constructing optimal experience for the hospitalized newborn through neuro-based music therapy. Front Hum Neurosci. 2015;9:487. doi: 10.3389/fnhum.2015.00487 26388762 PMC4558927

[45] Catapano F, Steinwurtzel R, Parravicini E, Wool C. A qualitative analysis of parents’ experiences while their neonates with congenital heart disease require intensive care. Front Pediatr. 2024;12:1425320. doi: 10.3389/fped.2024.1425320 39301041 PMC11410620

[46] Klawetter S, Gievers L, McEvoy CT, Nicolaidis C. NICU parent and staff advocacy to address parental mental health. Clin Pediatr (Phila). 2025;64(2):247–56. doi: 10.1177/00099228241260167 38853718 PMC11801483

[47] Laccetta G, Di Chiara M, De Nardo MC, Terrin G. Symptoms of post-traumatic stress disorder in parents of preterm newborns: A systematic review of interventions and prevention strategies. Front Psychiatry. 2023;14:998995. doi: 10.3389/fpsyt.2023.998995 36970259 PMC10032332

[48] Rambod M, Pasyar N, Mazarei Z, Soltanian M. The predictive roles of parental stress and intolerance of uncertainty on psychological well-being of parents with a newborn in neonatal intensive care unit: a hierarchical linear regression analysis. BMC pediatrics 2023:23(1), 607. doi: 10.1186/s12887-023-04420-4 38037025 PMC10691133

[49] Bozzette M. A review of research on premature infant-mother interaction. Newborn and Infant Nursing Reviews. 2007;7(1):49–55.

[50] Hartzell G, Shaw RJ, Givrad S. Preterm infant mental health in the neonatal intensive care unit: A review of research on NICU parent-infant interactions and maternal sensitivity. Infant Ment Health J. 2023;44(6):837–56. doi: 10.1002/imhj.22086 37815538

[51] Forcada-Guex M, Pierrehumbert B, Borghini A, Moessinger A, Muller-Nix C. Early dyadic patterns of mother-infant interactions and outcomes of prematurity at 18 months. Pediatrics. 2006;118(1):e107–14. doi: 10.1542/peds.2005-1145 16818525

[52] Balikci A, May-Benson TA, Sirma GC, Kardas A, Demirbas D, Aracikul Balikci AF, et al. The Homeostasis-Enrichment-Plasticity (HEP®) approach for premature infants with developmental risks: a pre-post feasibility study. J Clin Med. 2024;13(18):5374. doi: 10.3390/jcm13185374 39336861 PMC11432283

[53] Newnham CA, Milgrom J, Skouteris H. Effectiveness of a modified Mother-Infant Transaction Program on outcomes for preterm infants from 3 to 24 months of age. Infant Behav Dev. 2009;32(1):17–26. doi: 10.1016/j.infbeh.2008.09.004 19026450

[54] Lense MD, Shultz S, Astésano C, Jones W. Music of infant-directed singing entrains infants’ social visual behavior. Proc Natl Acad Sci U S A. 2022;119(45):e2116967119. doi: 10.1073/pnas.2116967119 36322755 PMC9659341

[55] Fancourt D, Perkins R. The effects of mother–infant singing on emotional closeness, affect, anxiety, and stress hormones. Music & Science. 2018;1. doi: 10.1177/2059204317745746

[56] Kobus S, Diezel M, Dewan MV, Huening B, Dathe A-K, Felderhoff-Mueser U, et al. Music therapy is effective during sleep in preterm infants. Int J Environ Res Public Health. 2021;18(16):8245. doi: 10.3390/ijerph18168245 34443994 PMC8391215

[57] Yakobson D, Gold C, Beck BD, Elefant C, Bauer-Rusek S, Arnon S. Effects of live music therapy on autonomic stability in preterm infants: a cluster-randomized controlled trial. *Children* (Basel, Switzerland). 2021: 8(11), 1077. doi: 10.3390/children8111077 34828790 PMC8618386

[58] Hugoson P, Haslbeck FB, Ådén U, Eulau L. Parental singing during kangaroo care: parents’ experiences of singing to their preterm infant in the NICU. Front Psychol. 2025;16:1440905. doi: 10.3389/fpsyg.2025.1440905 39968194 PMC11832525

[59] Smith AR, Hagan J, Walden M, Brickley A, Biard M, Rhee C, et al. The effect of contingent singing on infants with bronchopulmonary dysplasia in the neonatal intensive care unit. J Music Ther. 2023;60(1):98–119. doi: 10.1093/jmt/thac019 36592139

[60] Mimura K, Tomimatsu T, Endo M, Kimura T. Atypical preeclampsia with systemic lupus erythematosus and elevated soluble fms-like tyrosine kinase 1/placental growth factor ratio. J Obstet Gynaecol Res. 2021;47(12):4461–6. doi: 10.1111/jog.15055 34605122

[61] Spence MJ, Freeman MS. Newborn infants prefer the maternal low-pass filtered voice, but not the maternal whispered voice. Infant Behavior and Development. 1996;19(2):199–212.

[62] Mills M, Melhuish E. Recognition of mother’s voice in early infancy. Nature. 1974;252(5479):123–4. doi: 10.1038/252123a0 4420642

[63] Mai X, Xu L, Li M, Shao J, Zhao Z, deRegnier R-A, et al. Auditory recognition memory in 2-month-old infants as assessed by event-related potentials. Dev Neuropsychol. 2012;37(5):400–14. doi: 10.1080/87565641.2011.650807 22799760 PMC3399741

[64] Hirst D, Looze CD. Fundamental Frequency and Pitch. In R.-A. Knight & J. Setter (Eds.). The Cambridge Handbook of Phonetics. Cambridge University Press. 2021:336–61. doi: 10.1017/9781108644198.014

[65] Gerhard D. Pitch extraction and fundamental frequency: History and current techniques. Regina, Canada: Department of Computer Science, University of Regina. 2003:0–22.

[66] Titze IR. Physiologic and acoustic differences between male and female voices. J Acoustical Society of America. 1989;85(4):1699–707. doi: 10.1121/1.397959 2708686

[67] Coleman RO. A comparison of the contributions of two voice quality characteristics to the perception of maleness and femaleness in the voice. J Speech Hear Res. 1976;19(1):168–80. doi: 10.1044/jshr.1901.168 1271795

[68] Fant G. A note on vocal tract size factors and non-uniform F-pattern scalings. Speech Transmission Laboratory Quarterly Progress and Status Report. 1966;1:22–30.

[69] Latinus M, Taylor MJ. Discriminating male and female voices: differentiating pitch and gender. Brain Topogr. 2012;25(2):194–204. doi: 10.1007/s10548-011-0207-9 22080221

[70] Boren B, Roginska A, Gill B. Maximum averaged and peak levels of vocal sound pressure. In Audio Engineering Society Convention 135. Audio Engineering Society. 2013.

[71] Yiu EM-L, Yip PPS. Effect of noise on vocal loudness and pitch in natural environments: an accelerometer (Ambulatory Phonation Monitor) Study. J Voice. 2016;30(4):389–93. doi: 10.1016/j.jvoice.2015.05.016 26106071

[72] Restin T, Gaspar M, Bassler D, Kurtcuoglu V, Scholkmann F, Haslbeck FB. Newborn incubators do not protect from high noise levels in the neonatal intensive care unit and are relevant noise sources by themselves. Children (Basel). 2021;8(8):704. doi: 10.3390/children8080704 34438595 PMC8394397

[73] van der Stelt O, Belger A. Application of electroencephalography to the study of cognitive and brain functions in schizophrenia. Schizophr Bull. 2007;33(4):955–70. doi: 10.1093/schbul/sbm016 17363840 PMC2632335

[74] Pruitt T, Davenport EM, Proskovec AL, Maldjian JA, Liu H. Simultaneous MEG and EEG source imaging of electrophysiological activity in response to acute transcranial photobiomodulation. Front Neurosci. 2024;18:1368172. doi: 10.3389/fnins.2024.1368172 38817913 PMC11137218

[75] De Haan M. Infant EEG and event-related potentials. Psychology Press. 2013.

[76] Watanabe K, Hayakawa F, Okumura A. Neonatal EEG: a powerful tool in the assessment of brain damage in preterm infants. Brain Dev. 1999;21(6):361–72. doi: 10.1016/s0387-7604(99)00034-0 10487468

[77] Papatzikis E, Dimitropoulos K, Gerdes A, Tataropoulou K, Pasoudi E, Kyrtsoudi M, et al. Advancing EEG processing and analysis in neonatal music interventions: Introducing a specialized qEEG pipeline for premature infants in NICU. In: University of Kansas, Lied Center: Lawrence, KS, United States, 2024.

[78] Papatzikis E, Dimitropoulos K, Tataropoulou K, Kyrtsoudi M, Nika A. Advancing EEG processing and analysis in neonatal music interventions: Introducing a specialized qEEG pipeline for premature infants in the NICU. Music & Medicine. 2024;16(3):194.

[79] Papatzikis E, Dimitropoulos K, Gerdes A, Tataropoulou K, Pasoudi E, Kyrtsoudi M, et al. Advancing EEG processing and analysis in neonatal music interventions: Introducing a specialized qEEG pipeline for premature infants in NICU. In: Proceedings of the FIT’NG 2024 Conference, Baltimore, MD, USA, 2024.

[80] O’Toole JM, Boylan GB. NEURAL: quantitative features for newborn EEG using Matlab. arXiv preprint. 2017. doi: 10.48550/arXiv:1704.05694

[81] Pavlidis E, Lloyd RO, Mathieson S, Boylan GB. A review of important electroencephalogram features for the assessment of brain maturation in premature infants. *Acta paediatrica*. 2017: 106(9), 1394–408. 28627083 10.1111/apa.13956

[82] Pavlidis E, Lloyd RO, Livingstone V, O’Toole JM, Filan PM, Pisani F, et al. A standardised assessment scheme for conventional EEG in preterm infants. Clin Neurophysiol. 2020;131(1):199–204. doi: 10.1016/j.clinph.2019.09.028 31812080

[83] Britton JW, Frey LC, Hopp JL, Korb P, Koubeissi MZ, Lievens WE, et al. Electroencephalography (EEG): An introductory text and atlas of normal and abnormal findings in adults, children, and infants. 2016. 27748095

[84] Wallois F, Routier L, Heberlé C, Mahmoudzadeh M, Bourel-Ponchel E, Moghimi S. Back to basics: the neuronal substrates and mechanisms that underlie the electroencephalogram in premature neonates. Neurophysiol Clin. 2021;51(1):5–33. doi: 10.1016/j.neucli.2020.10.006 33162287

[85] Anvari F, Lakens D. Using anchor-based methods to determine the smallest effect size of interest. Journal of Experimental Social Psychology. 2021;96:104159.

[86] Althubaiti A. Sample size determination: A practical guide for health researchers. J Gen Fam Med. 2022;24(2):72–8. doi: 10.1002/jgf2.600 36909790 PMC10000262

[87] Kang H. Sample size determination and power analysis using the G*Power software. J Educ Eval Health Prof. 2021;18:17. doi: 10.3352/jeehp.2021.18.17 34325496 PMC8441096

[88] Hincu M-A, Zonda G-I, Stanciu GD, Nemescu D, Paduraru L. Relevance of biomarkers currently in use or research for practical diagnosis approach of neonatal early-onset sepsis. Children (Basel). 2020;7(12):309. doi: 10.3390/children7120309 33419284 PMC7767026

[89] Akoglu H. User’s guide to sample size estimation in diagnostic accuracy studies. Turk J Emerg Med. 2022;22(4):177–85. doi: 10.4103/2452-2473.357348 36353389 PMC9639742

[90] Dimitropoulos K. Evaluation framework of the process and implementation of the research protocol entitled: Study of brain activity in premature infants in the Neonatal Intensive Care Unit (NICU), investigating the fundamental frequency of the human voice during musical intervention (singing) and the potential differentiation caused by the infant’s familial relationship to the intervention provider (father, mother, male music therapist, female music therapist). Department of Music Science and Art, University of Macedonia. 2025.

[91] Harper RM, Hoppenbrouwers T, Bannett D, Hodgeman J, Sterman MB, McGinty DJ. Effects of feeding on state and cardiac regulation in the infant. Dev Psychobiol. 1977;10(6):507–17. doi: 10.1002/dev.420100604 598621

[92] Holditch-Davis D, Scher M, Schwartz T, Hudson-Barr D. Sleeping and waking state development in preterm infants. Early Hum Dev. 2004;80(1):43–64. doi: 10.1016/j.earlhumdev.2004.05.006 15363838

[93] White‐Traut RC, Nelson MN, Silvestri JM, Vasan U, Littau S, Meleedy‐Rey P, et al. Effect of auditory, tactile, visual, and vestibular intervention on length of stay, alertness, and feeding progression in preterm infants. Develop Med Child Neuro. 2002;44(2):91–7. doi: 10.1111/j.1469-8749.2002.tb00293.x 11848115

[94] Lehtonen J, Könönen M, Purhonen M, Partanen J, Saarikoski S. The effects of feeding on the electroencephalogram in 3‐ and 6‐month‐old infants. Psychophysiology. 2002;39(1):73–9. doi: 10.1111/1469-8986.3910073 12206297

[95] Schmidt Mellado G, Pillay K, Adams E, Alarcon A, Andritsou F, Cobo MM, et al. The impact of premature extrauterine exposure on infants’ stimulus-evoked brain activity across multiple sensory systems. Neuroimage Clin. 2022;33:102914. doi: 10.1016/j.nicl.2021.102914 34915328 PMC8683775

[96] Háden GP, Bouwer FL, Honing H, Winkler I. Beat processing in newborn infants cannot be explained by statistical learning based on transition probabilities. Cognition. 2024;243:105670. doi: 10.1016/j.cognition.2023.105670 38016227

[97] Trehub SE. Infants’ perception of musical patterns. Percept Psychophys. 1987;41(6):635–41. doi: 10.3758/bf03210495 3615157

[98] French C, Pinnock H, Forbes G, Skene I, Taylor SJC. Process evaluation within pragmatic randomised controlled trials: what is it, why is it done, and can we find it?-a systematic review. Trials. 2020;21(1):916. doi: 10.1186/s13063-020-04762-9 33168067 PMC7650157

[99] van ’t Westende C, Geraedts VJ, van Ramesdonk T, Dudink J, Schoonmade LJ, van der Knaap MS, et al. Neonatal quantitative electroencephalography and long-term outcomes: a systematic review. Dev Med Child Neurol. 2022;64(4):413–20. doi: 10.1111/dmcn.15133 34932822

[100] Bourel-Ponchel E, Gueden S, Hasaerts D, Héberlé C, Malfilâtre G, Mony L, et al. Normal EEG during the neonatal period: maturational aspects from premature to full-term newborns. Neurophysiol Clin. 2021;51(1):61–88. doi: 10.1016/j.neucli.2020.10.004 33239230

[101] Gao R. Interpreting the electrophysiological power spectrum. J Neurophysiol. 2016;115(2):628–30. doi: 10.1152/jn.00722.2015 26245320 PMC4752306

[102] Kapucu FE, Välkki I, Mikkonen JE, Leone C, Lenk K, Tanskanen JMA, et al. Spectral entropy based neuronal network synchronization analysis based on microelectrode array measurements. Front Comput Neurosci. 2016;10:112. doi: 10.3389/fncom.2016.00112 27803660 PMC5068339

[103] Vogelsang M, Vogelsang L, Diamond S, Sinha P. Prenatal auditory experience and its sequelae. Dev Sci. 2023;26(1):e13278. doi: 10.1111/desc.13278 35583318 PMC11164537

