# Supplementary material for: The father’s singing voice may impact premature infants’ brain more than their mother’s: A NICU single-arm exploratory study protocol and preliminary data on a singing and EEG framework based on the fundamental frequency of voice and kinship
Source: PLoS One. 2025 Aug 14;20(8):e0328211. doi: 10.1371/journal.pone.0328211 (PMC12352647; doi:10.1371/journal.pone.0328211)
Supplement: S1 Data — (PDF) [file pone.0328211.s001.pdf]

Version #1
10.03.2023

Studying newborns' brain activity in the NICU through a musical intervention: the role of fundamental frequency

Background

Premature newborns constitute a growing population in pediatric care. Prematurity is the leading cause of death in the first month of life for newborns and a major cause of long-term disability (Elder et al., 2009). However, due to advances in neonatal care, many premature newborns now survive. These advancements are taking place also in the field of research on the presence and characteristics of sound in neonatal intensive care units (NICUs), and more specifically, the effect of deliberately introduced sound in the form of music on the health and development of premature newborns (Neal & Lindeke, 2008).

Music, the art of combining acoustic frequencies and musical structures, creates a sophisticated blend of sounds. Research has shown that music benefits premature newborns receiving care in NICUs. Many studies on the effects of music on premature infants have found that it positively impacts brain development, stabilizes heart and respiratory rhythms, reduces the number of apnea and bradycardia events per day, and improves feeding and weight gain (Papatzikis et al., 2020; Anderson et al., 2018; Pineda et al., 2017; Lordier et al., 2019).

It is crucial to understand the distinction between sound, noise, and music in this context. Sound is a vibration (or set of vibrations) observed in a transmission medium (usually air) and has intensity (audibility), frequency (pitch), periodicity (repetition at regular intervals), and duration (Colvin et al., 2004). Sound can be modulated using patterns or left unformatted. Its intensity is measured in decibels (dB), and its frequency in Hertz (Hz) or cycles per second. Noise, a meaningless patterned or non-patterned sound, can be annoying and disturbing, and it may have negative psychological and biological effects on humans, particularly premature infants (Bremmer et al., 2003; Graven, 2000; Gray, 2000; Philbin, 2000). Music on the other hand, as organized sound (referred to music also from now on), produces positive biological and psychological reactions, contrasting with noise (Schwartz, 2003).

The concept of tone is fundamental in the analysis and study of the perception and influence of sound and music. A tone is a sound with a specific frequency and intensity (Helmholtz, 2009). Musical notes and the human voice are composed of a series of tones known as “partial” or “harmonic” tones (Helmholtz, 2009) (pp: 33-35). However, our ears recognize the foundation tone as the fundamental frequency of the note, while the remaining tones, double, triple, and so on, are defined as a single harmonic series (Helmholtz, 2009) and convey the connotation of the musical note. The unique distribution of the intensities of the combination of harmonic tones of each note produced by the instrument or voice, combined with the “way” (mechanical sequence) that the note begins to form in the first milliseconds of its execution, is what gives the sound its characteristic timbre and the timbre of each instrument and the human voice. For that matter, timbre is a property of sound that allows the human ear to distinguish between two sounds with the same intensity and frequency but originating from different sources.

Based on the mechanistic extensions resulting from the combination of the foundation tone and the harmonic tones, a specific acoustic frequency range of 500 to 1,000 Hz is observed in premature newborns born in the last trimester (25 to 37 weeks) (Glass, 1999; Roeser, 1996). Compared to the premature infants, what truly distinguishes the fetus is the attenuation of sound levels and the various frequency ranges with which it comes into contact. According to research, although auditory function begins around the 20th week, adult-like auditory limits (in terms of volume) do not mature until the 35th week of pregnancy (Gooding, 2010; Graven & Browne, 2008; Lasky & Williams, 2009). Simultaneously, the high-pass function filter of pregnancy tissues and fluids modulates any external stimulus before it reaches the fetus' auditory system, protecting the fetus from being overwhelmed by noise. As a result, the mother's uterus reduces frequency ranges above 500 Hz from 40 to 50 dB and frequency ranges below 500 Hz from 10 to 20 dB before reaching the fetus' auditory system. Consequently, when the intrauterine noise level exceeds the external noise level by approximately 10 dB, the fetus cannot hear speech or music in the frequency range below 500 Hz. In other words, the fetus can only hear extraneous sounds if the sound levels in the air exceed 60 dB (Gerhardt & Abrams, 2004) and are available in a specific range of basic sound frequencies. Lower frequency emergence in the uterine environment aids in focusing the fetus on the frequency range of speech (Standley, 2003). An adult man's speech has

a fundamental frequency range from 85 to 155 Hz, while an adult woman's speech has a frequency range from 165 to 255 Hz (Baken, 2000).

In this context, the mother's voice has a direct effect on the fetus, which provides insight into the fetus's ability to learn as it processes the sensory information derived from the mother's voice (Fifer & Moon, 1994). Prenatal exposure to auditory stimuli, particularly the mother's voice, is a prerequisite for postnatal language development (Nöcker-Ribaupierre, 1999; Maiello, 2004; McMahon et al., 2012; Wachman & Lahav, 2010).

What is the research idea?

According to research, full-term infants can distinguish between musical characteristics such as coherence and dissonance, differences in pitch, changes in melody, and specific acoustic ranges of tones (Lynch et al., 1992; Trainor & Heinmiller, 1998; Trainor & Trehub, 1992; Trainor & Trehub, 1993; Trainor & Zacharias, 1998; Tsang & Trainor, 2002). More specifically, studies have shown that newborn infants can: a) distinguish the words they heard repeatedly during their prenatal life from unfamiliar words (DeCasper & Fifer, 1980), b) prefer female voices to male ones, the mother's voice over other female voices, and voices of the same nationality over voices of other nationalities (Casper & Spence, 1986; Moon et al., 1993), and c) accurately distinguish between upward and downward intonation, even when both scales are sung by the same voice on the same vowel (Fernald, 1989).

While the above is true for full-term newborns, it has also been discovered that music calms premature newborns, reduces physiological instability, and increases brain activity in three-month-old infants (Schmidt et al., 2002).

However, when focusing on the physical characteristic of the fundamental frequency, there is no evidence for premature newborns related to the brain effect and cognitive processing of organized sounds - music. Based on the preceding research, and given that this element appears not to have been studied in the context of prematurity, particularly in the context of the acoustic/sound environment composed by the neonatal intensive care unit (NICU), we were led to the following research question:

Research Question: Does structured sound intervention from two different human profiles [(gender: male, female)] with a combined study element of the fundamental frequency result in a differentiated short-term electroencephalic effect footprint in

NICU infants? Furthermore, is there differentiation if their kinship (mother, father) with the newborn becomes a factor?

Research Hypothesis: H1: We hypothesize that when premature newborns are exposed to a musical intervention provided by their mother (high fundamental frequency – biological correlation) compared to the father (low fundamental frequency – biological correlation) or an unrelated male/female music therapist, they show statistically more visible short-term positive oscillatory differentiations (translated as developmental benefits in this context) in their brain activity.

H0: We assume that there is no interaction between the fundamental frequency and the human profile (mother, father, man, woman) in the infant's reactions, and that neither the fundamental frequency nor the human profile has a primary effect.

Methodology

This will be an exploratory/randomized control trial (RCT) study involving premature infants in the NICU.

According to our research questions and hypothesis, although our aim is to mainly and deeply study the effects of our exploratory intervention on a particular population of premature infants, we will move on to also include a control group in our data collection pipeline for the reasons explained further below.

Based on the above, premature newborns who meet the inclusion criteria stated below will be randomly enrolled in either an intervention or a control group. The former will be exposed to a structured musical intervention (which will include variable investigative conditions of silence and singing) for one week during their stay in the NICU, while the research team will take care for the latter group to not be exposed to any musical stimulation for the same amount of time. Electroencephalography (EEG) will be used to record the newborns' brain activity throughout their participation in the research study, while video data will be recorded, forming an auxiliary part to the EEG data collection process. These video recordings will focus on capturing the interaction between the infant and the music facilitator, who could be either a parent or a music therapist.

The rationale for incorporating video data is multifaceted. Firstly, the video recordings will complement the electroencephalogram (EEG) data in offering a comprehensive parallel view of the infants' neural and behavioral responses. Furthermore, the videos will serve as a tool for quality control by ensuring that the prescribed research protocol is uniformly followed by all facilitators, contributing to the scientific integrity of the research. Finally, the videos will allow for an examination of any synchrony or coordinated interactions between the infant and the facilitator, shedding light on phenotypical – not able to be measured by the EEG – aspects of the intervention.

The intervention will be delivered by four different profiles of individuals-facilitators: a male music therapist, a female music therapist, the mother, and the father of the newborn.

As mentioned above, although we will place more weight to the exploratory part of this study – as dictated by our research question and hypothesis – we will intentionally involve an RCT leg, as we believe that in basic exploratory research, control groups are essential not only for direct comparisons of treatment vs. no treatment, but also as reference points for more nuanced investigations to the exploratory parts of such studies. This includes comparing different types of interventions or various modalities within an intervention category, such as the different conditions embedded in our music intervention (i.e., singing versus complete silence) to the data received from the control group (i.e., silence in intervention group versus silence in control group). After all, the control group's role partly determines the research question the study answers. Therefore, the need to collect data from a control group is approached as default in our overall exploratory approach of our study, as it will allow us for a more comprehensive understanding of the intervention's effects.

More specifically, the inclusion of a control group in this mainly exploratory in character study is fundamental for the following compelling reasons:

- *Baseline Comparison:* The control group will establish a crucial baseline for comparison. Without it, distinguishing the specific effects of the music intervention would be challenging. This group will allow for a clear evaluation of whether the observed EEG changes are indeed due to the sound intervention characteristics or other unrelated factors.

- *Internal Validity:* The control group is key to ensuring the study's internal validity. It provides a means to determine that the changes in EEG patterns are genuinely attributable to the sound interventions and not confounded by other variables, such as the NICU environment or the natural developmental trajectory of the infants.

- *Ethical Considerations:* The inclusion of a control group ensures that all infants receive the standard of care. It also allows for further assessment of the music intervention's potential risks or benefits compared to this standard care, which is critical in conducting ethical research, especially in a sensitive setting like the NICU.

- *Scientific Rigor:* The control group significantly contributes to the scientific rigor of the study. It enables more precise and reliable statistical analyses and supports the drawing of valid conclusions.

Participants

The musical intervention will be conducted in the neonatal intesnive care unit of the Aglaia-Kyriakou Children's Hospital in Athens.

Newborns participating in the project must meet the following inclusion criteria:

- Born at ≤ 32 weeks of gestation
- Absence of auditory insufficiency in the 8th cranial nerve and excessive pathology in the brainstem
- Negative bilateral "transient evoked otoacoustic emissions," indicating unresponsiveness of the inner ear to an acoustic stimulus.
- Weighing 1000 grams or more
- In stable health conditions with no imminent risk of death
- Not sedated
- Absence of neonatal sepsis.
- No congenital, genetic, or chromosomal abnormalities.
- No nuclear jaundice.
- No maternal use of illicit drugs during pregnancy.
- No necessity for mechanical ventilation that creates excessive noise (i.e., high-frequency oscillatory ventilation).
- No need for sedative drug administration to the neonate.
- Absence of endocrine disorders (e.g., congenital hypothyroidism).

If a newborn is diagnosed with a disease or dysfunction during the study and the medical staff recommends their removal, they will be automatically excluded from the recruited sample.

Finally, to maintain the integrity of the study, restrictions on external musical stimulation, including singing to newborns during the admission period (especially for the control group) will be clearly communicated as non-compliance with the above guidelines and will necessitate the exclusion of the newborn from the study.

Sample Size - Power Analysis (Based on Minimum Effect Size of Interest)

In situations where an exact effect size is not derivable from prior research or pilot data – as our project's case – utilizing the Minimum Effect Size of Interest (MESI) is advantageous for guiding sample size estimation (Anvari & Lakens, 2021). This approach focuses on the smallest effect size that is considered practically or clinically significant within the specific context of our study. This effect size forms the foundation of our power analysis and by adopting a MESI, we ensure that our study is not only statistically robust but also resonates with clinical or practical relevance, thereby enhancing the applicability of our findings.

For this project the MESI was established at Cohen's d = 0.3. This determination was made by reviewing clinical guidelines – suggesting that even the smallest short-term positive neuroplastic change in this very sensitive population can make a difference – as well as relevant literature (i.e., Anvari & Lakens, 2021). In practice, this effect size reflects the minimal change in the primary outcome that would significantly impact patient care or treatment decisions.

Setting our alpha (Type I error) at 0.05 and power (Type II error) at 0.80, a power analysis – using the G*Power 3.1.9.7 software – suggested that 30 participants are needed to adequately power the study to detect this minimum effect size with desired statistical certainty.

**Effect Size Specification**	**Cohens'd 1988 = 0.3**
**Tool**	**G*Power 3.1.9.7**

**Analysis:**	**A priori: Compute required sample size**	
**Input:**	**Effect size f(V)**	**0,8**
	α err prob	0,05
	Power (1-β err prob)	0,95
	Number of groups	2
	Number of measurements	4
	Nonsphericity correction ε	1
**Output:**	Noncentrality parameter λ	19,2
	Critical F	2,7132271
	Numerator df	3
	Denominator df	84
	**Total sample size**	**30**
	Actual power	0,9621585

Ethics

The study has been approved by the Hospital's Scientific Council (IRB; protocol number: 09th/04-05-2023 (Θ: 9)), and it has been submitted for registration at the www.clinincaltrials.gov platform. The project will commence once all ethical considerations have been fulfilled and all members of the research team have been thoroughly informed about their roles according to this protocol.

Parents of newborns who meet the inclusion criteria will be invited to a one-on-one meeting with the research team after expressing interest in learning more about or participating in the study. This meeting will provide detailed information, both orally and in writing, on the intervention and data collection procedures, relevant research methodologies, selection, participation and withdrawal issues, confidentiality concerns, and study timeline. Specific clarifications regarding the non-invasive nature of our research tools (such as music and EEG) will be provided, and all pertinent questions will be addressed and answered.

More specifically, participating parents will be informed that their and their infants' involvement in the study is entirely voluntary and that they may opt out at any time without any impact on their current or future relationships with the researchers or other staff members of the Panagiotis & Aglaia Kyriakou Children's Hospital. It will be emphasized that all information provided by participants (parents, guardians, and

children) during the research period will be kept strictly confidential. In the event of opting out, their infant's and personal data will be promptly deleted.

Furthermore, parents will be briefed that all data collected during the study will be de-identified, psudonymized and assigned a unique identification number. Names or other information linking the participants to the collected data will not appear in any study analysis, report, or publication. The pseudonymized EEG and video recordings collected for analysis will be uploaded to a secure management platform in Norway, ensuring a high level of data security. Additionally, it will be clarified that no sensitive participant information, other than the numerical identifier, will be included in the transferred data to this secure platform. Parents will be also informed of their right to full access to the collected data.

After receiving, listening to, reading, and understanding all relevant research materials and procedures – as stated above – during the one-on-one meeting, parents will be asked to sign a consent form indicating their willingness to participate in the study.

Benefit/Risk Assessment

Benefits

In the realm of neonatal care, the anticipated benefits of this proposed study, centered on the investigation of structured sound intervention in the NICU, are indeed profound and multifaceted. With a focus on the interplay between fundamental frequencies and human profiles, such as gender and kinship, and their impact on brain activity in premature newborns, the study is poised to make significant contributions to the field.

One of the primary benefits is the enhanced understanding of neurodevelopmental processes. By delving into how premature infants process various auditory stimuli, this research is set to enrich scientific knowledge considerably. The findings are expected to offer vital insights into the specific neural responses elicited by sound interventions, paving the way for more targeted and effective therapeutic strategies. These strategies are anticipated to be instrumental in promoting optimal brain development in preterm infants, a critical aspect of their overall health and well-being.

Moreover, the potential for improvement in neonatal care is substantial. Should the music intervention prove successful, it could be integrated into the standard care

protocols within NICU settings, representing a significant stride forward in neonatal healthcare. This non-invasive intervention could bolster the neurodevelopmental progress of premature infants, potentially enhancing not only their cognitive abilities but also their emotional and overall developmental outcomes. The integration of such interventions in routine care could, therefore, mark a pivotal shift towards more holistic and effective neonatal healthcare practices.

Furthermore, the innovative approach adopted in this study, which combines EEG analysis with behavioral observations, is set to establish new precedents in neonatal research. This comprehensive framework promises to elucidate the effects of various stimuli on infant development in a more holistic manner, thereby contributing to a richer, more nuanced understanding of neonatal care and development.

The significance of these benefits is underscored by a robust body of scientific literature, which highlights the biological and psychological advantages of musical interventions in NICU settings. Studies such as those by Bieleninik, Ghetti, and Gold (2016) and Loewy et al. (2013) have consistently indicated that music can offer substantial benefits compared to the absence of musical stimuli. These benefits are not only considerable but also well-documented, further reinforcing the potential impact and importance of this research in advancing neonatal care practices and outcomes.

Risks and Mitigation Strategies

In undertaking this research, it's crucial to recognize and address the potential risks associated with the study's non-invasive nature while also outlining the strategies to mitigate these concerns.

Overstimulation of Infants: The risk of overstimulation due to sound exposure stands as a primary concern. To address this, meticulous monitoring of sound levels will be enforced, ensuring they remain within established safe limits. A dedicated dB dosimeter will be employed for precise measurement and control, while the timing of the intervention will be strategically planned to guarantee adequate rest periods for the infants, thereby minimizing any potential disturbance. Additionally, the study will utilize a specifically designated, quiet, and adequately equipped room within the NICU floor. This setting will not only take place to preserve the calmness of the infants but also to provide an environment that prevents any undue stress on the facilitators. By maintaining a controlled and serene atmosphere, the facilitators will be able to deliver

the intervention without inadvertently increasing volume levels, thereby safeguarding against potential overstimulation of the infants.

Stress to Infants Due to Handling: Addressing the stress to infants due to handling, particularly for EEG setup, is another vital aspect. The research team is committed to ensuring that all procedures involve minimal handling and always are conducted in the comforting presence of caregivers. At this point, it is essential to note that the application of EEG electrodes, as assessed in various studies, does not inherently lead to discomfort or stress, either physical or psychological. This is supported by literature, such as the findings presented by Nathalie et al. (2018), which show no significant risk or discomfort associated with EEG applications in similar settings. However, to further mitigate any potential stress and ensure the safety of the procedure, specialists in applied neurological neonatology will be part of the research team. These professionals, with extensive training and experience in applying EEG electrodes to infants, will ensure that the procedure adheres to the highest standards of safety, in line with national and international guidelines.

Unforeseen Medical Complications: In the occurrence of unforeseen medical complications or adverse reactions, the study protocol includes immediate cessation of the intervention and prompt medical evaluation of the infant. This prompt response and thorough evaluation ensure the safety and well-being of the participants throughout the study.

Preparedness and Planned Measures

Study Preparedness

The research team embarking on this study has meticulously prepared to address the multifaceted demands of the applied protocol, ensuring preparedness, scientific rigor, and adherence to ethical standards.

Comprehensive Training of Staff and Facilitators: As a result, central to our preparedness efforts is the comprehensive training of all staff and facilitators. This encompasses a broad spectrum of skills and knowledge areas crucial for the study's success. Specifically, it includes the nuanced handling of preterm infants, effective delivery of the musical intervention, and technical proficiency in operating EEG equipment. This training will ensure that music therapists, caregivers, neonatologists,

and pediatric neurologists involved in the study are well-equipped to fulfill their roles with expertise and care.

Establishment of Protocols for Emergency Situations: Building on this foundation of well-trained personnel, we have established robust protocols for emergency situations. These protocols, which align with the existing hospital and NICU emergency procedures, are designed to provide swift and coordinated responses to any medical emergencies that might arise during the study. All staff and team members will be thoroughly briefed on these procedures, ensuring everyone is prepared to act quickly and effectively, safeguarding the safety of the participants.

Regular Monitoring and Evaluation: Further enhancing our preparedness, regular monitoring and evaluation will be a constant throughout the study. This will be meticulously overseen by the responsible neonatologist, a key member of our research team. Such continuous oversight is not just about maintaining the study's integrity; it's crucial for ensuring the ongoing safety and well-being of the infant participants. This proactive approach to monitoring underscores our commitment to the highest standards of care throughout the research process.

Measures to Ensure Scientific Rigor and Ethical Conduct

Parallel to these rigorous preparatory measures, we are equally committed to ensuring scientific rigor and ethical conduct in every aspect of the study. To begin with, the study protocol will undergo a thorough ethical review and approval process, aligning with the highest international standards for research involving human subjects. This rigorous review process is essential for affirming the ethical soundness and compliance of our study, setting a solid foundation for the research to proceed. Therefore, we have already been prepared to apply for ethical approval at the IRB of the participating to this study hospital, as well as to the Norwegian Regional Committee for Medical and Healthcare Research Ethics (REK). For more information on the ethics related to this project, please read the designated related part - Ethics - of this research proposal.

Furthermore, an integral part of our ethical commitment is reflected in our comprehensive informed consent process. Parents of participating infants will be engaged in an extensive dialogue, where they will be provided with detailed and transparent information about the study through a well-designed information leaflet. This will include the project's objectives, potential risks, benefits, and their rights,

particularly their ability to withdraw from the study at any time. Ensuring that participants' rights and autonomy are respected is paramount and is a cornerstone of our ethical approach.

Finally, to maintain the study's focus and align it with its objectives, regular team meetings and audits will be a staple. These sessions will not only serve as opportunities to review progress and tackle any issues but also reinforce adherence to the study protocol and ethical guidelines. Periodic audits will further cement this commitment, ensuring that our study remains on track, adhering to established protocols, and upholding the highest standards of scientific inquiry and ethical conduct.

Data Privacy and Confidentiality

Data privacy and confidentiality are paramount in a study involving sensitive data such as EEG recordings and videos of premature infants. To safeguard this aspect, rigorous data management protocols will be implemented.

First of all, all collected EEG and Video data will be de-identified (as of their meta-data) at the source. Specifically for the EEG data, during the export procedure from the EEG unit’s software platform in the EDF file format, a specialized EDF de-identifier/anonymizer software will be implemented on site, to extract and erase all meta-data pointing to personal identification information found natively (i.e., embedded automatically from the EEG unit) in the EDF file. This new de-identified EDF file (containing the EEG data) will then be assigned a unique identification number. With this step, pseudonymization will be achieved, separating personal data from direct identifiers in the files. The linkage between the unique identification number of the file and the personal data of the participant will only be possible through the usage of a key identifier.

A similar process will be followed for the meta-data of the Video files, although manually. Especially for the Video files, as faces and voices will be clearly shown and heard when used and analyzed (i.e., this cannot be avoided due to the protocol needs explained above), locking and encryption (both in transit and at rest) will be implemented to them as a second layer of security to protect them from unauthorized access.

When the above steps have been completed, both the EEG and Video de-identified pseudonymized data will be securely stored in the TSD Norwegian data management platform, a high-security online platform for sensitive research data. This platform ensures that no sensitive participant information, other than the assigned numerical identifier, is included in the transferred data. The key linking numerical identifiers to identifiable participant information will be encrypted with a password, and securely stored separately and accessible only by the Principal Investigator. Upon completion of the project, all identifiers and keys will be destroyed. Access to this data will be strictly limited to authorized personnel only. In case of withdrawal, all personal data related to the participant will be promptly deleted.

Justifiability of the project relative to risks and benefits

The proposed research project on studying newborns' brain activity in the NICU through a musical intervention presents a comprehensive and well-structured approach aimed at investigating the effects of structured sound intervention on premature infants' brain development. Therefore, the project's justifiability relative to risks and benefits can be presented by the following consideration points:

1. Methodology

- The study employs a randomized controlled trial (RCT) design, which is considered the gold standard for evaluating the efficacy of interventions. This design allows for comparisons between treatment and control groups, enhancing the reliability of the findings.

- The inclusion criteria for participants are clearly defined, ensuring that only infants meeting specific criteria are enrolled in the study. This helps maintain the homogeneity of the sample and increases the internal validity of the results.

- The use of electroencephalography (EEG) to measure brain activity, supplemented by video recordings, provides comprehensive data to assess both neural and behavioral responses to the intervention. This multi-modal approach strengthens the study's validity and allows for a more nuanced understanding of the intervention's effects.

2. Ethical Considerations:

- The study protocol emphasizes the importance of obtaining informed consent from parents, ensuring that they fully understand the nature of the study, potential risks, and benefits. This reflects a commitment to respecting participants' autonomy and rights.

- Measures are in place to protect participant confidentiality and privacy, including de-identification of data and secure storage protocols. This demonstrates a commitment to ethical conduct and safeguarding participants' sensitive information.

3. Potential Benefits:

- The study aims to enhance understanding of neurodevelopmental processes in premature infants and explore the efficacy of a non-invasive intervention (musical stimulation) in promoting optimal brain development. If successful, the intervention could be integrated into standard care protocols, potentially improving outcomes for premature infants in NICU settings.

- The project has the potential to contribute to scientific knowledge in the field of neonatal care and developmental psychology, paving the way for more targeted and effective therapeutic strategies. This could have far-reaching implications for improving the long-term health and well-being of premature infants.

4. Risk Mitigation Strategies:

- The study incorporates measures to mitigate potential risks, such as overstimulation of infants and stress due to handling. These include monitoring sound levels, providing adequate rest periods, and ensuring procedures involve minimal handling by experienced personnel. Additionally, protocols are in place to respond promptly to any unforeseen medical complications.

In light of these considerations, the benefits of the project appear to outweigh the potential risks or disadvantages to the participants. The rigorous methodology, ethical considerations, and risk mitigation strategies demonstrate a commitment to conducting the study in a responsible and ethical manner. Therefore, the project can be considered justifiable relative to the risks participants may be exposed to, as the potential benefits of advancing scientific knowledge and improving neonatal care outcomes are substantial and reasonable.

Research Design, Tools, and Data Analysis

Design

Preterm newborns who meet the inclusion criteria will be exposed to a specific musical intervention (in terms of content and exposure timing) four times in a calendar week – once per day. This repeated measures design is strategically chosen to ensure the inclusion of all music facilitators delivering the music intervention once within a single week, thereby minimizing the potential for data contamination from confounding variables. By conducting the intervention in the proposed way, we can observe and record the immediate and short-term neurophysiological effects of the musical stimuli on the infants' brains, while ensuring that the data reflects the true impact of the intervention, free from external variables that might arise from a more prolonged or sporadic schedule. Furthermore, the specific exposure will facilitate a comprehensive understanding of any cumulative or evolving effects of the intervention across consecutive sessions, allowing for a detailed analysis of the EEG baselines, and in result providing invaluable insights into the neurodevelopmental responses of the preterm newborns to the musical intervention over a consistent period. For this reason, the facilitators' order of intervention delivery for each newborn will be changed weekly to achieve better randomization and to avoid potential confounding variables due to a set order of intervention delivery.

Most importantly, the specific design's structure allows for a thorough examination of the stability and variability of the EEG baselines, crucial for interpreting the intervention's effectiveness. Regular monitoring of the EEG baselines before and after each sung session – as will be detailed below – will enable us to distinguish between the natural developmental trajectory of the newborns' brain activity and the specific changes induced by the musical intervention, not only enhancing in this way the reliability and validity of the findings, but also providing a robust foundation for better understanding the potential therapeutic benefits of music in neonatal care.

In addition to the points mentioned above, it's important to note that the study protocol will be conducted in a dedicated room, largely isolated from the ambient noise typically found in the rest of the NICU. This aspect of the study design is crucial for two main reasons:

Firstly, ensuring the comfort and safety of the participating newborns requires adherence to the recommended standards for the NICU sound environment. Therefore, the importance of a well-managed acoustic environment in reducing stress and discomfort for our participating infants is of paramount importance. Such a consideration has been consistently highlighted both for clinical and research settings alike (European Standards of Care for Newborn Health; EFCNI, Sizun, J., Hallberg, B., et al., 2018), and a sound-controlled room within the NICU seems to be the only viable option for achieving the necessary level of sound management without disrupting other clinical operations. In this context, when considering the operational sound, as outlined by White, Smith, Shepley, et al. (2013), the combination of continuous background sound and operational sound should not exceed an hourly L_{eq} of 45 dB and an hourly L_{10} of 50 dB, both A-weighted slow response, with transient sounds or Lmax not exceeding 65 dB. Our study, with sound exposure lasting less than 3 minutes per hour, will strictly adhere to these guidelines. However, the transient nature of singing, distinct from the continuous and better-controlled sound pressure levels typically found in continuous speech, might lead to brief instances where we slightly exceed these thresholds by about 15 dB. According to evidence, even professional singers, and more so untrained individuals like the parents participating in our study, often produce transient peak Sound Pressure Levels (SPLs) of approximately 78-79 dB when singing softly (Boren, Roginska & Gill, 2013). Therefore, a dedicated room will significantly aid in ensuring the correct application and viability of these recommendations. Secondly, recent research, including that by Restin et al. (2021), suggests that incubators can be significant sources of noise, particularly in the open context of the NICU floor. As a result, a quiet and controlled NICU environment is crucial for the success of our study, avoiding this way external sound challenges that could compromise the integrity of the protocol and the accuracy of data collection. A dB measuring device will be stationed outside the incubator in this quiet environment during the intervention to ensure all the above.

As far as the musical intervention is concerned, this will take place in two main singing stages complemented by three distinctive blocks of silence, before, between and after each sung session.

- * 180 seconds of silence

- 1st singing stage: Signing a specific tone at 440 Hz, repeated ad libitum for a total of 60 seconds.
- * 180 seconds of silence
- 2nd singing stage: Singing an original song suitable for the NICU environment in terms of structure, acoustic information composition, and volume level (dB), with a total duration of 90 seconds.
- * 180 seconds of silence

We introduce silence before and after each singing stage of the intervention in order to firstly record a baseline brain activity before and after the singing process, but also to facilitate a return to the baseline of brain activity in between the two singing stages, allowing in result for a more accurate assessment of the effects of each individual singing stage.

In regards to the singing stages, as stated above, all facilitators will be asked to sing at the same distant location from the incubator, maintaining in this way consistent spatial sound parameters of stimulus delivery. For the first stage of the singing part, each facilitator will be given a tuning fork (a forked acoustic component that produces sound upon impact and quickly silences) tuned to the musical note A (440 Hz) and asked to sing based on this the musical note A (440 Hz) as best as they can. All facilitators will need to repeat the specific note ad libitum over 90 seconds at this stage. The tuning fork has been chosen to be used as it is a safe acoustic accessory for accurately reproducing the tone the facilitator needs to hear as an acoustical template when inserting it into their ear, ensuring that the newborn will not be able to hear it or be affected by it.

For the second singing stage, each facilitator will need to sing a complex acoustic stimulus with organized sounds (song), specifically created and composed for the study by a professional musician (a member of the research team). This song will have a unique structure and acoustic information composition adhering to the know scientific evidence related to infants' sound and music perception capabilities. The song will last 90 seconds and feature a soft melodic line, gentle rhythm, simple harmonies, and soft vocal timbre (Keith, Russell, & Weaver, 2009; Shoemark, 1999). Its tempo will be approximately 75 bpm, corresponding to the heart rate of a pregnant woman, which has

been found not to cause overstimulation or stress in newborns (Keith, Russell, & Weaver, 2009).

All parents of newborns who meet the inclusion criteria for the study will be trained in the intervention and will rehearse with the professional musician (a member of the research team) until they have mastered adequately the two stages of singing.

EEG Tool and Data Collection

Electroencephalography (EEG) is an electrobiological imaging tool commonly used in medicine and research. The electroencephalogram measures changes in electrical dynamics resulting from the formation of electrical dipoles during nerve stimulation. The EEG signal consists of several brain waves that reflect electrical activity in the brain based on electrode placement and function in adjacent brain areas.

EEG-based changes in response to musical interventions have become a popular method for investigating brain function in patients with various brain and mental health disorders, as well as in healthy individuals. Recently, this method has been also applied to premature newborns and full-term infants (Wu J et al., 2013).

High-amplitude EEG (aEEG) is an increasingly used approach in NICUs, with proven clinical value for monitoring newborn brain function. In our study, we will collect data from premature newborns using the aEEG method, as described below:

- The electrical activity of the neonatal brain in the intervention group will be recorded using a four-electrode aEEG device for five consecutive minutes before, during, and after the intervention. The Burdjalov scoring system will be then employed for EEG coding, interpretation, and statistical analysis (Burdjalov, Baumgart & Spitzer, 2003).

The Burdjalov scoring system

The Burdjalov scoring system is based on the analysis of high-amplitude EEG recordings. The system examines the presence and frequency of abnormal brain waves and assigns a severity score based on their presence and frequency. The four component variables of the aEEG record are evaluated, classified, and scored using the scoring system proposed in Table 1 below. Each recording's overall score is calculated by

adding the component subscores. The minimum possible overall score is 0, and the maximum is 13.

- Continuity
- Presence or absence of "cycling"
- Amplitude (in µV) of the lower border
- Bandwidth span

Table 1.

Score	Continuity	Cycling	Amplitude of Lower Border	Bandwidth Span and Amplitude of Lower Border
0	Discontinuous	None	Severely depressed (<3 µV)	Very depressed: low span (≤15 µV) and low voltage (5 µV)
1	Somewhat continuous	Waves first appear	Somewhat depressed (3–5 µV)	Very immature: high span (>20 µV) or moderate span (15–20 µV) and low voltage (5 µV)
2	Continuous	Not definite, somewhat cycling	Elevated (>5 µV)	Immature: high span (>20 µV) and high voltage (>5 µV)
3		Definite cycling, but interrupted		Maturing: moderate span (15–20 µV) and high voltage (>5 µV)
4		Definite cycling, noninterrupted		Mature: low span (<15 µV) and high voltage (>5 µV)
5		Regular and mature cycling		

Statistical Data Analysis

We aim to investigate the effects of musical intervention on the Burdjalov scoring system of premature infants, while considering the role of different facilitators and the fundamental frequencies of their voices. In this case, we believe that a mixed-effects regression model may be the most appropriate statistical model.

The mixed-effects regression model allows us to account for the variability of subjective repetitive measurements, as well as that created between subjects to differentiate the effects of facilitators. It also enables us to include multiple predictor variables (such as the presence or absence of the musical intervention, the facilitator's profile, and their fundamental frequency) while controlling for potential confounders.

More specifically, we will use the Burdjalov score as the dependent variable, with the following predictor variables included:

Group: whether the infant was in the intervention or control group
Time: the point in time when the baseline (intervention measurement) was obtained
Facilitator: the person who provides the intervention (male music therapist, female music therapist, mother, or father)
Fundamental frequency: the fundamental frequency of the facilitator providing the intervention.

Additionally, we could include other covariates, such as the infant's age, gestational age, and any other relevant demographic or clinical variables that we believe will contribute to a better understanding of our measurements.

References

Anderson, D. E., & Patel, A. D. (2018). Infants born preterm, stress, and neurodevelopment in the neonatal intensive care unit: might music have an impact? Developmental Medicine & Child Neurology, 60(3), 256-266.

Anvari, F., & Lakens, D. (2021). Using anchor-based methods to determine the smallest effect size of interest. *Journal of Experimental Social Psychology*, *96*, 104159.

Baken, R. J., & Orlikoff, R. F. (2000). *Clinical measurement of speech and voice*. Cengage Learning.

Bieleninik, Ł., Ghetti, C., & Gold, C. (2016). Music therapy for preterm infants and their parents: A meta-analysis. Pediatrics, 138(3), e20160971. https://doi.org/10.1542/peds.2016-0971

Boren, B., Roginska, A., & Gill, B. (2013, October). Maximum averaged and peak levels of vocal sound pressure. In *Audio Engineering Society Convention 135*. Audio Engineering Society.

Bremmer, P., Byers, J. F., & Kiehl, E. (2003). Noise and the premature infant: Physiological effects and practice implications. Journal of Obstetric,Gynecologic, and Neonatal Nursing, 32, 447–454.

Cassidy, J. W., & Standley, J. M. (1995). The effect of music listening on physiological responses of premature infants in the NICU. *Journal of music therapy*, *32*(4), 208-227.

Colvin, M., McGuire, W., & Fowlie, P. W. (2004). ABC of preterm birth: Neurodevelopmental outcomes after preterm birth. British Medical Journal, 329(7479), 1390–1393.

DeCasper, A. J., & Fifer, W. P. (1980). Of human bonding: Newborns prefer their mother’s voices. Science, 208, 1174–1176.

EFCNI, Sizun, J., Hallberg, B., et al. (2018). European Standards of Care for Newborn Health: Management of the acoustic environment.

Elder, D.E., A. Wong & J.M. Zuccollo. 2009. Risk factors for and timing of death of extremely preterm infants. Aust. NZ J. Obstet. Gynaecol. 49: 407–410.

Fernald, A. (1989). Intonation and communicative intent in mothers’ speech to infants: Is the melody the message? Child Development, 60, 1497–1510.

Fifer, W. P., & Moon, C. M. (1994). The role of the mother’s voice in the organization of brain function in the newborn. (Supplemental material). Acta Paediatrica, 83(s397), 86–93. DOI: 10.1111/j.16512227.1994. tb 13270.x.

Gerhardt, K. J., & Abrams, R. M. (2004). Fetal hearing: Implications for the neonate. In M. NöckerRibaupierre (Ed.), Music therapy for premature and newborn infants (pp. 21–32). Gilsum, NH: Barcelona Publishers.

Glass, P. (1999). The vulnerable neonate and the neonatal intensive care environment. In G. B. Avery, M. A. Fletcher, & M. G. McDonald (Eds.), Neonatology: Pathophysiology and management of the newborn (5th ed., pp. 91–108). Philadelphia: Lippincott.

Gooding, L. F. (2010). Using music therapy protocols in the treatment of premature infants: An introduction to current practices. The Arts in Psychotherapy, 37, 211–214.

Graven, S. N. (2000). Sound and the developing infant in the NICU: Conclusions and recommendations for care. Journal of Perinatology, 20(8, Pt. 2), S88–S93.

Graven, S., & Browne, J. (2008). Auditory development in the fetus and infant. Newborn and Infant Nursing Review, 8(4), 187–193.

Gray, L. (2000). Properties of sound. Journal of Perinatology, 20(8, Pt. 2), S6–S11.

Helmholtz, H. L. (2009). On the Sensations of Tone as a Physiological Basis for th Theory of Music. Cambridge University Press.

Keith, D. R., Russell, K., & Weaver, B. S. (2009). The effects of music listening on inconsolable crying in premature infants. *Journal of music therapy*, *46*(3), 191-203.

Lasky, R. E., & Williams, A. L. (2009). Noise and light exposures for extremely low birth weight newborns during their stay in the neonatal intensive care. Pediatrics, 123, 540–546

Lordier, L., Meskaldji, D. E., Grouiller, F., Pittet, M. P., Vollenweider, A., Vasung, L., & Hüppi, P. S. (2019). Music in premature infants enhances high-level cognitive brain networks. Proceedings of the National Academy of Sciences, 116(24), 12103-12108.

Lynch, M. P., & Eilers, R. E. (1992). A study of perceptual development for musical tuning. Perception & Psychophysics, 52, 599–608

Maiello, S. (2004). On the meaning of prenatal auditory perception and memory for the development of the mind: A psychoanalytical perspective. In M. Nöcker-Ribaupierre (Ed.), Music therapy for premature and newborn infants (pp. 85–96). Gilsum, NH: Barcelona Publishers.

McMahon, E., Wintermark, P., & Lahav, A. (2012). Auditory brain development in premature infants: The importance of early experience. Annals of the New York Academy of Sciences, 1252, 17–24.

Moon, C., Cooper, R. P., & Fifer, W. P. (1993). Two-day-olds prefer their native language. Infant Behavior and Development, 16, 495–500.

Nathalie, M., Mathur, A. M., Jain, S., Vesoulis, Z. A., & Zempel, J. M. (2018). Long term electroencephalography in preterm neonates: Safety and quality of electrode types. Clinical neurophysiology, 129(7), 1366-1371.

Neal, D., & Lindeke, L. (2008). Music as a nursing intervention for preterm infants in the NICU. *Neonatal Network*, *27*(5), 319-327.

Nöcker-Ribaupierre, M. (1998). Short- and long-term effects of the maternal voice on the behaviors of very low birth weight infants and their mothers as a basis for the bonding process. In R. R. Pratt & D. Grocke (Eds.), MusicMedicine 3: Expanding horizons (pp. 153–161). Victoria: University of Melbourne.

Papatzikis, E., Zeba, F., Särkämö, T., Ramirez, R., Grau-Sánchez, J., Tervaniemi, M., & Loewy, J.(2020). Mitigating the Impact of the Novel Coronavirus Pandemic on Neuroscience and Music Research Protocols in Clinical Populations. Frontiers in Psychology, 11, 2160.

Philbin, M. K. (2000). The influence of auditory experience on the behavior of preterm newborns. Journal of Perinatology, 20(8, Pt. 2), S77–S87.

Pineda, R., Guth, R., Herring, A., Reynolds, L., Oberle, S., & Smith, J. (2017). Enhancing sensory experiences for very preterm infants in the NICU: an integrative review. Journal of perinatology, 37(4), 323-332.

Roeser, R. J. (1996). Audiology desk reference. New York: Thieme.

Schmidt, L. A., Trainor, L. J., & Santes, D. L. (2002). Development of frontal encephalogram (EEG) and heart rate (ECG) responses to affective musical stimuli during the first 12 months of post-natal life. Brain and Cognition, 52, 27–32

Schwartz, F. J. (2003). Music and sound effect on perinatal brain development and the premature baby. In J. V. Loewy (Ed.), Music therapy in the neonatal intensive care unit (2nd ed., pp. 9–19). New York: The Louis & Lucille Armstrong Music Therapy Program, Beth Israel Medical Center.

Shoemark H. Singing as the foundation for multi-modal stimulation. In: Pratt R, Grocke D, editors. Music medicine 3, music medicine and music therapy: expanding horizons. Victoria, Australia: The University of Melbourne, 1999: 140–52

Standley, J. M. (2003a). Music therapy with premature infants. Research and developmental interventions. Silver Spring, MD: American Music Therapy Association.

Trainor, L. J., & Heinmiller, B. M. (1998). The development of evaluative responses to music: Infants prefer to listen to consonance over dissonance. Infant Behavior & Development, 21, 77–88.

Trainor, L. J., & Trehub, S. E. (1992). A comparison of infant's and adult's sensitivity to Western musical structure. Journal of Experimental Psychology. Human Perception and Performance, 18, 394–402.

Trainor, L. J., & Trehub, S. E. (1993). Musical context effects in infants and adults: Key distance. Journal of Experimental Psychology. Human Perception and Performance, 19, 615–626.

Trainor, L. J., & Zacharias, C. A. (1998). Infants prefer higher-pitched singing. Infant Behavior & Development, 21, 799–806.

Tsang, C. D., & Trainor, L. J. (2002). Spectral slope discrimination in infancy: Sensitivity to socially important timbres. Infant Behavior & Development, 25, 183–194.

Wachman, E. M., & Lahav, A. (2010). The effects of noise on preterm infants in NICU.

White, R. D., Smith, J. A., Shepley, M. M., et al. (2013). Recommended standards for newborn ICU design, eighth edition. *Journal of Perinatology, 33*(Suppl 1), S2-S16.

Wu J, Zhang J, Ding X, Li R, Zhou C. The effects of music on brain functional networks: a network analysis. Neuroscience. 2013; 250:49-59

Restin, T., Gaspar, M., Bassler, D., Kurtcuoglu, V., Scholkmann, F., & Haslbeck, F. B. (2021). Newborn incubators do not protect from high noise levels in the neonatal intensive care unit and are relevant noise sources by themselves. Children, 8(8), 704.
