# Supplementary material for: The father’s singing voice may impact premature infants’ brain more than their mother’s: A NICU single-arm exploratory study protocol and preliminary data on a singing and EEG framework based on the fundamental frequency of voice and kinship
Source: PLoS One. 2025 Aug 14;20(8):e0328211. doi: 10.1371/journal.pone.0328211 (PMC12352647; doi:10.1371/journal.pone.0328211)

[redacted]

Advancing EEG Processing and Analysis in Neonatal Music Interventions: Introducing a Specialized qEEG Pipeline for Premature Infants in NICU

[redacted]

Efthymios Papatzikis[1]; Kyriakos Dimitropoulos[2]; Aubrianna Gerdes[3]; Kassandra Tataropoulou[4]; Elena Pasoudi[6]; Maria Kyrtsoudi[5]; Angeliki Nika[4]

NICU and Infant Brain Development

- Infants in the NICU face developmental obstacles due to prematurity, comorbidities, and the stressful environment of the NICU noise (Seassau et al., 2023).
- Measuring/Monitoring infant brain development during hospitalization can track potential abnormalities in maturation (Hellstrom-Westas & Rosén, 2006). If an abnormality is detected, early intervention can begin to reduce the potential long-term detriments.

Music-Based Interventions (MBIs) in the NICU

- In utero, the infant is exposed to many sound stimuli, typically low frequency due to the amniotic fluid sound filter (Gerhardt & Abrams, 2000); however, maternal-related sounds as well as exteral to the uterus sound stimuli are still audible (Mikulis, Inder & Erdei, 2021).
- Due to premature birth, proper in-uterus (brain) development is disrupted (Ment & Vohr, 2008)
- Since sound is present during early development it is genrally hypothesized that appropriate auditory stimuli in the NICU like music could foster developmental progression (McMahon et al., 2012)
- It has been shown that MBIs can support widespread neural function including the regulation of the autonomic nervous system with heart rate and respiration stabilization, weight gain and sleep (Haslbeck, 2017).
- Furthermore, MBIs elicit widespread connectivity in the premature infant brain evident by fMRI measurement (Lordier et al, 2019).

EEG and MBIs in the NICU for Infant Brain Development

- Neuroimaging-Guided MBIs have started to emerge in the context of the NICU, paving the way to a more robust application of music in this sensitive context (Papatzikis, 2024)
- Electroencephalography (EEG) is one of the available non-invasive methods to measure an infant's brain electrical activity.
- This relatively new approach (for neonates) is commonly used to diagnose infants with seizure disorders, sleep apnea, or brain conditions like tumors/infections. It can also be used to examine the infant brain development in general or neural response to a stimulus such as sound and music (Giraud et al., 2023).

Current Infant EEG and MBIs Pre-processing/Processing Knowledge

- Infant EEG often includes an abundance of artifacts due to excessively unpredictable, non-controlled movement (Georgieva et al., 2020)
- Infant EEG profile is different from that of adults' (Britton et al., 2016)
- Infants can only tolerate short durations of EEG, limiting the total amount of data (Debnath, 2020). This creates difficulty in determining the brain signal from what is unrelated.
- Several Infant EEG Pre-processing/Processing pipelines have been developed so far: HAPPE, BEAPP, MADE, NEAR (see refs for more info)

Statements to Consider

in Premature Infant EEG and MBIs Pre-processing/Processing Pipeline

- NICU Related
  - The noisy and unpredictable NICU environment can pose extra EEG recording issues compared to the term infant/lab based recording (i.e., non isolated environment; tratment of acute symtomps/clinical crisis; other equipment electrical contamination etc).
- MBI Related
  - Music is a complex stimulus, which must be tailored to the infant's needs and development level. For this, it can provide another level of complexity when incorporated with a sensitive data collection method such as EEG. (e.g., possible recording times vs actual/needed stimulation times) in the NICU, when compard to adults
- EEG Related
  - Premature infants' EEG profile is different from term infants' and adults' EEG due to structural and functional differences.
  - For premature infants, differentiation/complexity of signal is conversely analogous to the gestation age. Most importantly continuity/discontinuity; frequency domain specificities; connectivity/interhemispheric coherence change acording to the gestation age

Knowledge Gap

in Premature Infant EEG and MBIs Pre-processing/Processing Pipeline

- No specific EEG preprocessing/processing pipeline exists for MBIs taking into consideration the specific EEG profile of the premature infants

Research Objective

- To develop and validate a standardized semi-automated pre-processing/processing pipeline for EEG data collected from infants in the NICU on a short active stimulus, ensuring high-quality quantitative EEG (qEEG) end-user data.

EEG Methods

- MatLab 2024a and EEGLab 2024.02 was used to analyze the EEG signal
- *Preprocessing*: Custom MatLab code was created – EEGLab readymade functions
- *Processing*: the NEURAL code by Toole & Boylan (2017) was adapted to fit the needs of the processing analysis
- The EEG data collection sampling rate was 512 Hz
- Condition samples analyzed according to protocol = 100 samples
- EEG samples (based on sampling rate) =
  - One session duration 690 secs / four sessions duration 2760 secs x 512 Hz sampling rate = 1.413.120 samples per infant
  - 5 Infants analyzed = 7.065.600 samples in total

Data Collection Protocol

Protocol Overview
•**Participants:** 5 Premature infants
•**Duration:** Four consecutive days, once per day
•**Site:** A dedicated, quite NICU room

Session Structure

-180 Seconds of Silence (Silence1)
- Purpose: Establish baseline EEG activity.

-1st Singing Stage (Note A)
- **Duration:** 60 seconds
- **Activity:** Singing a specific tone at 440 Hz, repeated ad libitum.
- **Purpose:** Assess infant response to a consistent auditory stimulus

-180 Seconds of Silence (Silence2)
- Purpose: Monitor recovery and return to baseline EEG activity.

-2nd Singing Stage (Lullaby)
- **Duration:** 90 seconds
- **Activity:** Singing an original song suitable for the NICU environment, considering structure, acoustic composition, and volume level (dB).
- **Purpose:** Evaluate infant response to a more complex auditory stimulus.

-180 Seconds of Silence (Silence3)
- **Purpose:** Final observation of recovery and return to baseline EEG activity.

Facilitator Variation
•**Music Facilitators**:
- Mother
- Father
- Male Music Therapist
- Female Music Therapist

•**Voice Characteristics**:
- High Fundamental Frequency (Female)
- Low Fundamental Frequency (Male)

•**Kinship Level**:
- **Close Relationship:** Mother, Father
- **No Relationship:** Male and Female Music Therapists

About Us; References ········>

Results - Outcome

The configured semi-automated preprocessing and processing qEEG pipeline presented here, applied to short-duration, controlled auditory stimuli like music in the NICU, delivers robust signal quality and performing stability.

Benefits

•This statistically validated and meticulously developed pipeline, focused on the specific NICU neurodevelopmental context, ensures more accurate and reliable EEG readings in premature infants, enabling new possibilities for developmental EEG analysis on auditory stimuli in the NICU.
•With the integration of the NEURAL software—specifically adapted for short music/sound stimuli across different conditions—and custom MATLAB scripts designed to enhance the preprocessing pipeline according to the American Clinical Neurophysiology Society Guidelines (2013; 2024) for standardized EEG interpretation in premature infants, this pipeline becomes a valuable tool in neonatal neuroimaging.
•The semi-automated nature of its application (i.e., EEGLab tools + specially configured MATLAB scripts) enhances efficiency, enabling repeatable and objective EEG analysis results rather than subjective interpretations.

Future Clinical Applicability

•Personalized Medicine in the NICU
•Broader Use of MBIs
•Predictive Modeling and Longitudinal Studies

Limitations

•The customization of MATLAB scripts, although enhancing automation and efficiency, introduces a level of complexity that may require specialized knowledge and resources for implementation in different clinical settings.
•The validation of this pipeline was conducted on a relatively small sample size, which may affect the generalizability of the findings.
•Future research should aim to address these limitations by expanding the pipeline's applicability to a broader range of stimuli, simplifying its implementation, and validating it across larger and more diverse populations.

Step 1: Formatting

Import raw (512Hz) data .edf file to EEGLab for high resolution preprocessing

Check and Adjust (if needed) electrode layout; 8 unipolar channels - usually Fp1, Fp2, C3, C4, T3, T4, 01, 02

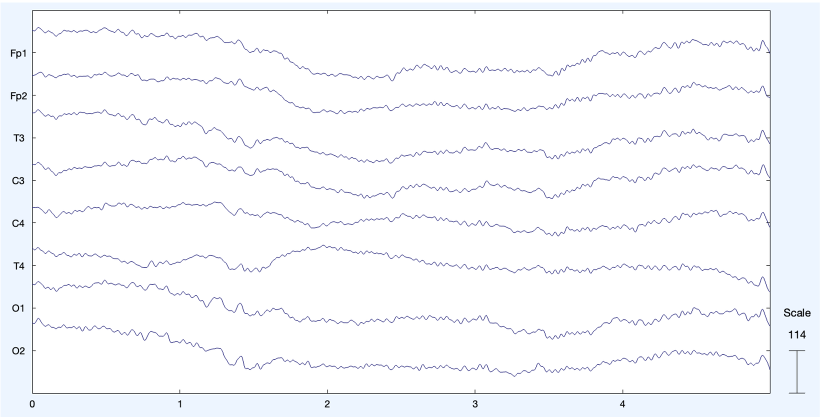

Segment data into study conditions for individual file preprocessing

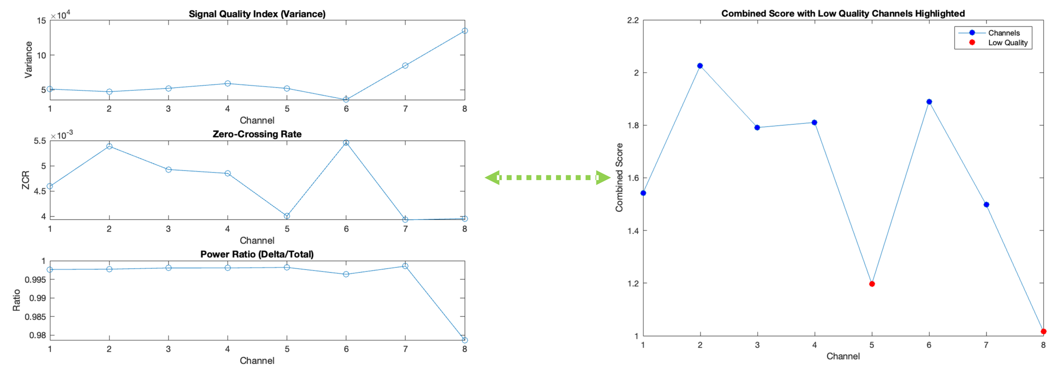

Step 2: Preprocessing

FIR: Remove frequencies outside of 0.1-30 Hz

CleanLine: Remove residual electrical interference

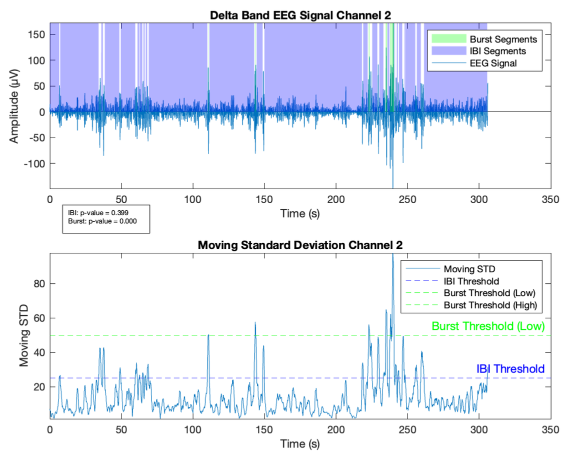

Custom code to calculate channel quality - Average of Signal Quality Index (SQI), Zero Crossing Rate (ZCR) and Power in frequency bands

Custom code to calculate individual channel discontinuity and Inter Burst Intervals (IBI)

Remove low-quality channels according to Average SQI, ZCR and Power Ratio

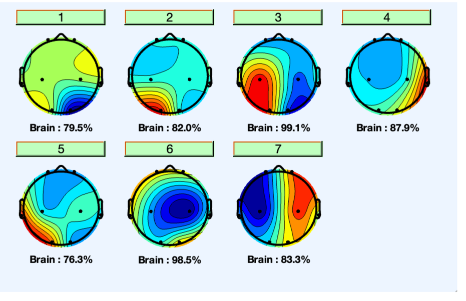

Perform Independent Component Analysis (ICA) and then Artifact Subspace Reconstruction (ASR)

Performed Statistical SNR and Entropy analysis to establish the most efficient line of steps according to the specific protocol and intervention

Step 3: Preprocessing Conclusion

Re-reference data

Interpolate channels

Resample data to 64 Hz

Create 8-channel bipolar montage

Create .mat file for qEEG processing with the NEURAL software

Copyrighted Image

NEURAL Software for EEG Analysis

Feature Extraction

Amplitude Features | Spectral Features | Connectivity Features | Burst and Inter-burst Interval Features

Clinical Application

Signal to Noise Ratio and Entropy Statistical Calculation of two Preprocessing Paths

The preprocessing path SNR_ICAASR (M = 0.239, SD = 0.090) produced a significantly higher mean SNR compared to SNR_ASRICA (M = 0.192, SD = 0.090), t(2037) = -2.971, p = 0.003, with an estimated difference of -0.048. Both preprocessing methods significantly improved SNR compared to the baseline SNR_Before (M = 0.002, SD = 0.008), with SNR_ICAASR being the most efficient.

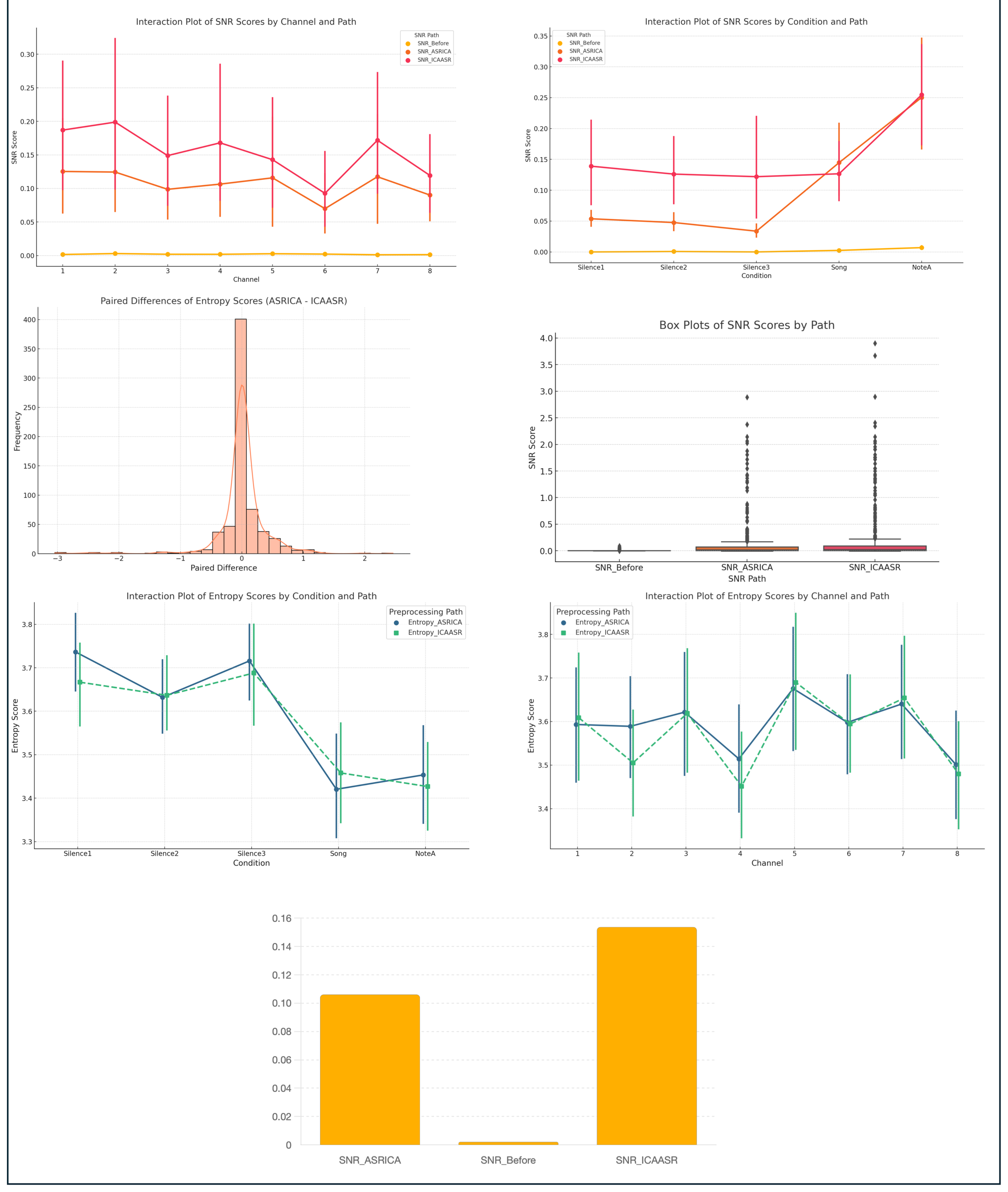

Supplement: S3 Data — (PDF) [file pone.0328211.s003.pdf]
